# Modulation of Synaptic Plasticity in the Cortex Needs to Understand All the Players

**DOI:** 10.3389/fnsyn.2017.00002

**Published:** 2017-02-01

**Authors:** Claire N. J. Meunier, Pascal Chameau, Philippe M. Fossier

**Affiliations:** ^1^Institut de Neurosciences Paris-Saclay (NeuroPSI), UMR 91197 CNRS-Université Paris-SaclayParis, France; ^2^Swammerdam Institute for Life Sciences, Center for NeuroScience, University of AmsterdamAmsterdam, Netherlands

**Keywords:** prefrontal cortex, serotonin, dopamine, LTP and LTD, neuromodulation

## Abstract

The prefrontal cortex (PFC) is involved in cognitive tasks such as working memory, decision making, risk assessment and regulation of attention. These functions performed by the PFC are supposed to rely on rhythmic electrical activity generated by neuronal network oscillations determined by a precise balance between excitation and inhibition balance (E/I balance) resulting from the coordinated activities of recurrent excitation and feedback and feedforward inhibition. Functional alterations in PFC functions have been associated with cognitive deficits in several pathologies such as major depression, anxiety and schizophrenia. These pathological situations are correlated with alterations of different neurotransmitter systems (i.e., serotonin (5-HT), dopamine (DA), acetylcholine…) that result in alterations of the E/I balance. The aim of this review article is to cover the basic aspects of the regulation of the E/I balance as well as to highlight the importance of the complementarity role of several neurotransmitters in the modulation of the plasticity of excitatory and inhibitory synapses. We illustrate our purpose by recent findings that demonstrate that 5-HT and DA cooperate to regulate the plasticity of excitatory and inhibitory synapses targeting layer 5 pyramidal neurons (L5PyNs) of the PFC and to fine tune the E/I balance. Using a method based on the decomposition of the synaptic conductance into its excitatory and inhibitory components, we show that concomitant activation of D1-like receptors (D1Rs) and 5-HT_1A_Rs, through a modulation of NMDA receptors, favors long term potentiation (LTP) of both excitation and inhibition and consequently does not modify the E/I balance. We also demonstrate that activation of D2-receptors requires functional 5-HT_1A_Rs to shift the E-I balance towards more inhibition and to favor long term depression (LTD) of excitatory synapses through the activation of glycogen synthase kinase 3β (GSK3β). This cooperation between different neurotransmitters is particularly relevant in view of pathological situations in which alterations of one neurotransmitter system will also have consequences on the regulation of synaptic efficacy by other neurotransmitters. This opens up new perspectives in the development of therapeutic strategies for the pharmacological treatment of neuronal disorders.

## Introduction

The prefrontal cortex (PFC) plays an important role in the processing of cognitive functions such attention, memory, decision making and more specifically in working memory (Fuster, 2001; Miller and Cohen, 2001). Its ability to integrate and process sensory information is ensured by a neuronal network receiving numerous afferent connections from sensory areas and making efferent connections to motor area and subcortical area (Miller and Cohen, 2001; Uylings et al., 2003). The higher-order brain functions performed by the PFC are supposed to rely on rhythmic electrical activity generated by neuronal network oscillations. Indeed, cortical networks are permanently active and their activity patterns, depending on the wakefulness or sleep states, are characterized by slow oscillations of the neuronal membrane potential (so-called up and down states) during slow wave sleep and by fluctuations around a depolarized level (persistent up state) during REM sleep and wakefulness (Steriade et al., 2001). Membrane potential fluctuations, the above described phase transitions between up and down state, can be spontaneously generated by local (surrounding) cortical synaptic connections. The up state is initiated by the recruitment of neurons through recurrent excitation and is regulated by local feedback inhibition, pyramidal and non-pyramidal neurons receiving strong barrages of synaptic input that cause membrane depolarization and action potential firing (up state). The down state is characterized by neuronal hyperpolarization, a reduction in synaptic bombardment and a reduction of action potential activity (Sanchez-Vives and McCormick, 2000; Timofeev et al., 2000; Compte et al., 2003; Shu et al., 2003a). This transition to the down state may result from synaptic depression or the buildup of intracellular calcium and sodium concentrations that activate potassium channels in neurons of the local network (Contreras et al., 1996; Sanchez-Vives and McCormick, 2000; Timofeev et al., 2000). These cortical network rhythms are determined by a precise balance between excitation and inhibition that results from the coordinated activities of recurrent excitation and feedback and feedforward inhibition (Shu et al., 2003b; Haider et al., 2006). The initiation of the up state is characterized by an increase in excitatory conductance followed by an increase in inhibitory conductance. Following onset and during the up state, excitatory and inhibitory conductances remain proportionally increased and dynamically balanced (Haider et al., 2006). This balanced activity keeps neurons at a noisy and elevated level of depolarization near firing threshold and prevents aberrant network activity. In addition to being spontaneously generated (Sanchez-Vives and McCormick, 2000; Timofeev et al., 2000; Shu et al., 2003b), both transitions from up to down and from down to up can be triggered by sensory input and activation of afferent inputs (Anderson et al., 2000; Petersen et al., 2003; Shu et al., 2003a; MacLean et al., 2005; Rigas and Castro-Alamancos, 2007).

Within cortical networks, interneurons are in charge of the dynamic adjustment of the level of excitation, and their role in the maintenance of a dynamic balance between excitation and inhibition balance (E-I balance) is essential in cortical function (Shu et al., 2003b). The E-I balance is a value that reflects the activity of neuronal network at one time (Haider and McCormick, 2009). A proper E-I balance is essential for physiological processes such as sensory perception, short term memory, long term memory and development (Saghatelyan et al., 2001; Egorov et al., 2002; Monier et al., 2003; Zhang et al., 2011). Alterations in maintaining the E-I balance have been observed in several pathologies such as epilepsy and autism. For instance, an increase in GABAergic transmission leading to a change in excitation has been observed in epileptic tissue (Cossart et al., 2001). In the case of autism, the E-I balance appears to vary in the direction of greater excitation (Rubenstein and Merzenich, 2003; Powell, 2004). These pathologies, currently considered as polygenic and multifactorial disorders, are characterized by a disruption of normal cortical connections with aberrant synaptic function and disorganized neurotransmitter interactions (Stephan et al., 2009). For instance, dysfunctions of dopaminergic, glutamatergic and serotoninergic systems have been associated with the pathophysiology of schizophrenia and major depression (Marek, 2007; Kantrowitz and Javitt, 2012). Although the pathophysiology of schizophrenia has been associated with alterations of the dopaminergic system (Creese et al., 1976; Meisenzahl et al., 2008), others suggest that the use of antipsychotic drugs acting at both the serotonergic and dopamine (DA) systems must be considered (Meltzer et al., 2004; Newman-Tancredi et al., 2007; Jones and McCreary, 2008; Newman-Tancredi, 2010; Meltzer and Massey, 2011; Newman-Tancredi and Kleven, 2011). Several studies have suggested that an imbalance between DA and serotonin (5-HT) systems could be the cause of adverse side effects observed during treatment of some pathologies (Borah and Mohanakumar, 2007; Carta et al., 2007; Navailles et al., 2010). These observations offer the possibility of a new therapeutic approach which consists in combining drugs that target several receptors to neuromodulators in the treatment of schizophrenia and major depression in order to reassess a proper E-I balance required for harmonious brain functions.

Synaptic plasticity is a fundamental property of the brain which allows the nervous system to adapt its response to experience. In this review article, we first briefly address the general mechanisms of synaptic plasticity leading to long term potentiation (LTP) or Depression of excitatory and inhibitory synapses and we introduce the notion of E-I balance. We also illustrate how the method we developed to determine the E-I balance offers the possibility to study simultaneously the plasticity of excitatory and inhibitory inputs on a layer 5 pyramidal neuron (L5PyN). The second part of this review article highlights our recent findings regarding the cooperation between serotoninergic and dopaminergic systems within the PFC in the orientation of synaptic plasticity towards potentiation or depression.

## How to Learn: Synaptic Plasticity

Synaptic plasticity is a fundamental property of neuronal circuits which endows the nervous system the ability to adapt its responses to a changing environment (sensory information). It confers the brain the capacity to adapt its behavior and to anticipate and control executive functions (Feldman, 2009). First postulated by Hebb (1949), the concept of synaptic plasticity was substantiated by Bliss and Lomo (1973) who demonstrated that, in hippocampus, a high frequency stimulation (HFS) of excitatory synapses induced a persistent enhancement of excitatory neurotransmission, the so called LTP. This extensively studied synaptic property is supposed to be the basic mechanism of learning and memory. It is initiated within a short period of time (a few seconds), it is stable and persistent after induction (for several weeks *in vivo*), it is initiated by frequencies of stimulation (theta burst) similar to the neuronal rhythmic activity observed in cortical networks engaged in various information processing tasks. Long term depression (LTD), the opposite of LTP, corresponds to a long-lasting decrease of synaptic efficacy (several hours) in response to low frequency stimulations (Dudek and Bear, 1992; Bear and Malenka, 1994).

These two forms of long-lasting synaptic plasticity do not oppose each other but rather are considered to be complementary in memory formation. For instance, it has been shown, in hippocampus, that LTP and LTD have respective roles in encoding spatial information (Kemp and Manahan-Vaughan, 2004). LTD is necessary to acquire a new object location in a new environment while LTP is associated with spatial exploration. Other studies have suggested that LTD could contribute to the acquisition of new information by weakening previously strengthened synapses and by preventing interferences with this new incoming information (Etkin et al., 2006; Nicholls et al., 2008; Malleret et al., 2010).

Most of the studies conducted over the past decades relate to the plasticity of excitatory synapses and it is rather recently that attention has been focused on the plasticity of inhibitory synapses. In the following chapters, we will summarize the knowledge on these two aspects of long-term synaptic plasticity.

### Plasticity of Excitatory Synapses

#### LTP

First discovered in hippocampus in the mid-eighties, LTP of excitatory synapses is generally considered as being a process requiring the activation of post (or pre) synaptic NMDA receptors and an increase in intracellular calcium that leads to an increase in synaptic efficacy after HFS of presynaptic terminals (Malenka and Nicoll, 1999). The most generally described mechanism to induce LTP is the activation of post-synaptic AMPA and NMDA receptors following a massive release of glutamate at the synaptic cleft induced by HFS of the presynaptic afferents. The activation of post-synaptic AMPA receptors results in a strong depolarization of the post-synaptic element and removes the magnesium blockade of the NMDA receptors (Kleckner and Dingledine, 1988; Burnashev et al., 1992; Calabresi et al., 1992) allowing cations to flow through the activated NMDA receptors. The high calcium permeability of NMDA receptors results in a calcium influx into the post-synaptic compartment that leads to changes in synaptic strength through a well described mechanism involving the activation of the calmodulin-dependent protein kinase II (CaMKII) and the subsequent phophorylation of AMPA receptors (increased open probability) and/or the trafficking of AMPA receptors to the post-synaptic membrane. Other kinases have been reported to be involved in the trafficking of AMPA receptors. Recent studies have shown that LTP can be induced at the presynaptic level either through a modulation of pre-synaptic NMDA receptors or through neuromodulators (NO, BDNF) released from the post-synaptic site acting at the presynaptic level. Following a theta-burst stimulation in hippocampus, McGuinness et al. (2010) have shown that action potentials at the synapse evoke a calcium influx through voltage-dependent calcium channel (VDCC) that induces glutamate release which can activate presynaptic NMDA receptors. This together with the removal of the Mg^2+^ block of NMDA receptors by depolarization results in an increase in intracellular calcium concentration and an enhanced glutamate release. Other mechanisms have been proposed to be responsible for the induction of LTP at excitatory synapses. The best characterized is the regulation of synaptic strength by retrograde messengers such as nitric oxide (NO). As shown in hippocampus, calcium ions entering the post-synaptic element through NMDA receptors activate NO synthase to produce NO from L-arginine. NO diffuses back to the presynaptic element and activates guanylate cyclase (GC) leading to the synthesis of cGMP and the activation of PKG and an increase in glutamate release (Costa et al., 2011). More recent studies reported that post-synaptic protein synthesis can also play a role in the regulation of the pre-synaptic mechanisms involved in persistent forms of LTP (Johnstone and Raymond, 2013). It has also been suggested that postsynaptic activation of the translation promotor mTOR complex 1 (mTORC1) results in the synthesis of the retrograde messenger BDNF and an enhancement of neurotransmitter release (Henry et al., 2012). Although NMDA receptors have a key role in the induction of LTP, it has been demonstrated in the visual cortex and in hippocampus (Johnston et al., 1992) that other players can trigger the calcium signal responsible for LTP induction. Grover and Teyler (1994) have shown that, in hippocampus, pairing presynaptic stimulation with post synaptic depolarization or with action potential firing results in a post synaptic calcium influx through L-type VDCC responsible for the induction of LTP. Other forms of NMDA-independent LTP have also been reported in rodent cortex that requires the activation of metabotropic glutamate receptors (Wilsch et al., 1998; Huemmeke et al., 2002).

#### LTD

The induction of LTD of excitatory synapses is mainly determined by the magnitude of the post-synaptic increase in intracellular calcium concentration (Mulkey and Malenka, 1992). A smaller increase in intracellular calcium concentration evoked by the activation of NMDA receptors will lead to the activation of a signaling cascade involving phosphatases such as calcineurin and PP1 (Lisman and Zhabotinsky, 2001) which results in the de-phosphorylation of synaptic AMPA receptors and their internalization causing a reduction of synaptic efficacy. Other forms of LTD involve the activation of metabotropic glutamate receptors followed by an increase in intracellular calcium concentration. Depending on the receptor subtype different signaling pathways are engaged. In pyramidal neurons from the neocortex (Czarnecki et al., 2007), mGluR1 receptors activation causes an increase in intracellular calcium that leads to the activation of protein kinase C (PKC) and AMPA receptors internalization. It has also been suggested that activation of mGluR5 or mGluR1 receptors, respectively in *striatum* and in *nucleus accumbens*, results in an increase in post-synaptic calcium concentration and in the synthesis of endocanabinoïds (eCBs) which activate presynaptic CB1R to decrease glutamate release (Chevaleyre et al., 2006). Finally another form of LTD has been described in visual cortex where LTD is induced by coincident activation of presynaptic NMDA auto-receptors and of CB1R, activated by retrograde signaling of eCBs (Sjöström et al., 2003).

### Plasticity of Inhibitory Synapses

Post synaptic changes of GABAergic neurotransmission can be explained by: (i) a modulation of GABA receptors activity by protein kinases such as PKC, CaMKII or by phosphatases such as calcineurine (Kittler and Moss, 2003; Houston et al., 2009); (ii) trafficking of GABAA receptors at the synapse (Saliba et al., 2007; Arancibia-Cárcamo and Kittler, 2009; Castillo et al., 2011). As for excitatory synapses, modulations of postsynaptic calcium concentration are supposed to play a role in the induction of inhibitory synaptic plasticity. It has been demonstrated in hippocampus that LTP of inhibitory synaptic transmission is induced by an increase in post-synaptic calcium concentration resulting from the activation of GABAB receptors and the subsequent synthesis of IP3 leading to the release of calcium from the IP3-sensitive calcium store (Gaiarsa and Ben-Ari, 2006). It has also been demonstrated that, in the case of heterosynaptic plasticity, different sources of calcium (VGCC, ionotropic NMDA receptors, metabotropic mGluR1 glutamate receptors) contribute to the increases in post-synaptic calcium concentration responsible for changes in synaptic efficacy (Goldberg et al., 2003; Nyíri et al., 2003; Topolnik et al., 2005). This variety of post-synaptic calcium sources allows a precise control of calcium signaling and a fine tuning of synaptic plasticity (Camiré and Topolnik, 2012).

The best characterized form of presynaptic plasticity of GABAergic synapse involves the activation of presynaptic receptors by retrograde messengers such as: eCBs, BDNF and NO. eCB-mediated synaptic plasticity has been described in several brain regions such as hippocampus (Chevaleyre and Castillo, 2003), amygdala (Marsicano et al., 2002; Azad et al., 2004), striatum (Adermark and Lovinger, 2009), visual cortex (Jiang et al., 2010) and cerebellum (Pitler and Alger, 1992; Llano et al., 1994). The induction of eCB-mediated plasticity is initiated by glutamate release from presynaptic terminals which induces the post-synaptic synthesis of eCBs. eCBs retrogradely activate CB1R located on presynaptic afferents from GABAergic interneurons (heterosynaptic iLTD) to decrease GABA release through a signaling cascade which inhibits the cAMP/protein kinase A (PKA) pathway (Heifets and Castillo, 2009). Whereas eCBs induce i-LTD, other retrograde messengers such as BDNF and NO can induce i-LTP. It has been shown in visual cortex and hippocampus (Lu, 2003; Gubellini et al., 2005; Sivakumaran et al., 2009) that the activation of NMDA receptors and/or VGCC and the subsequent increase in intracellular calcium concentration in the post synaptic element can result in the synthesis of BDNF or NO which increases GABA release (Nugent et al., 2007).

## Balanced Neuronal Networks

Neurons in the cerebral cortex consist of a majority of excitatory (glutamatergic) pyramidal neurons (75%–80%) making synaptic contacts both locally (local network) and over long distances (across distinct cortical areas) and of inhibitory (GABAergic) interneurons (25%–20%) making extensive local connections. Within a given local cortical network, neurons receive inputs from neighboring neurons which form recurrent excitatory and feedforward and feedback inhibitory circuits (Peters and Kara, 1985; White, 1989; Abeles, 1991; Markram et al., 2004). The great diversity of interneurons, in terms of their connectivity and their functional properties, allows them to control cortical excitation leading to a dynamic equilibrium between excitation and inhibition (Shu et al., 2003b). This dynamic control of excitation that regulates cortical network activity is performed through feedback and feedforward inhibition (Isaacson and Scanziani, 2011). Feedforward inhibition occurs when excitatory afferents activate first interneurons, prior to activating principal neurons, and results in a reduced excitation of the target neuron. This well described mechanism (Isaacson and Scanziani, 2011) contributes to the weakening of excitatory inputs. Feedback inhibition takes place when active principal neurons excite inhibitory interneurons which, in a feedback loop, inhibit principal neurons themselves (for instance, see Silberberg and Markram, 2007; Berger et al., 2010) to ensure the stability to the network.

It is remarkable to note that despite the diversity of neuronal networks, the relative contribution of excitatory and inhibitory input conductance to a given neuron is dynamically maintained at comparable values across different cortical layers and different cortical areas to guarantee a proper balance between excitation and inhibition (E-I balance). The E-I balance, determined as the ratio between excitatory and inhibitory input conductance onto a neuron evoked by an electrical stimulation of afferents (*in vitro*) or by sensory stimuli (*in vivo*), is maintained at approximately 20% excitation and 80% inhibition (Le Roux et al., 2006; Monier et al., 2008; Lucas-Meunier et al., 2009; Zhang et al., 2011; Xue et al., 2014; den Boon et al., 2015). This dynamic E-I balance is thought to result from the coordinated activities of direct and recurrent excitation and (feed-forward and feedback) inhibition and reflects the activity of neuronal networks at a given moment (Haider and McCormick, 2009; Isaacson and Scanziani, 2011). A proper balance between excitatory and inhibitory inputs onto cortical neurons is essential to maintain the stability of cortical networks in order to perform cognitive functions such as memory and sensory information processing. For instance, it determines proper cortical network rhythms responsible for higher order cognitive functions (Shu et al., 2003b; Haider et al., 2006). It is also essential for physiological processes such as short term memory and long term memory (Saghatelyan et al., 2001; Egorov et al., 2002). Cortical responses elaborated by L5PyNs depend on the balance between excitatory and inhibitory inputs perceived (Borg-Graham et al., 1998; Wehr and Zador, 2003). Balanced excitatory and inhibitory neurotransmission also appears to be fundamental in the fine tuning of neuronal responses to specific sensory features.

It is conceivable to think that any alterations of excitation or inhibition will generate aberrant information processing in the cortex (Haider and McCormick, 2009) and will lead to pathological situations. Indeed, disturbances in the E-I balance are associated with a broad spectrum of neuropsychiatric and neurological diseases, such as autism, schizophrenia and epilepsy (Cobos et al., 2005; Lewis et al., 2005; Rubenstein, 2010). It has been observed that, in epilepsy, an altered maturation of chandelier cells (interneurons) interferes with the activity of pyramidal neurons (Marco et al., 1996), leading to an unbalanced E-I ratio (Cossart et al., 2005). It is also hypothesized that, in schizophrenia, a dysfunction of NMDA receptors (Coyle, 2006) changes the level of excitation and consequently the E-I balance (Kehrer et al., 2008). Finally, a dysregulation of the E-I balance has also been observed in autism (Rubenstein and Merzenich, 2003; Powell, 2004; Rippon et al., 2007), Rett syndrome (Dani et al., 2005) and Tourette syndrome (Singer and Minzer, 2003).

Given that neuronal networks require a proper E-I balance to perform cognitive functions, how does network stability deal with synaptic plasticity? In other words, how to learn within dynamically balanced networks? The learning process might not only be considered not only as a gain or a loss of efficacy at a given synapse but also as dynamic changes of both excitatory and inhibitory synaptic strength. This homeostatic maintenance of excitation and inhibition balance may be a determining factor in the regulation of neuronal input-output function and information processing (learning) within a given neuronal network (Daoudal and Debanne, 2003; Staff and Spruston, 2003; Marder and Buonomano, 2004). Although the effects of concerted regulation of excitation and inhibition are still poorly understood, it has been suggested that balanced or imbalanced changes in synaptic strength of excitatory and inhibitory inputs have a different impact on the neuronal input-output function and consequently on neuronal firing. Indeed the two parameters, threshold and gain (rate of change or sensitivity of the input-output function), that characterize the probability of a neuron to fire action potentials as a function of stimulus intensity can be modulated by the ratio between excitation and inhibition. By combining a computational model with experimental data obtained in hippocampus, Carvalho and Buonomano (2009) have shown that imbalanced plasticity of excitatory synaptic inputs affects the threshold while the E-I balance affect the gain allowing neurons to optimize their information processing.

The method we use to determine the E-I balance is based on the continuous measurement of evoked postsynaptic currents and the decomposition of the conductance dynamics into its excitatory and inhibitory components (Monier et al., 2003, 2008). This continuous and simultaneous determination of both excitatory and inhibitory inputs also offers the possibility to determine the changes in excitatory and inhibitory synaptic strength occurring after applying a high frequency stimulus evoking long-term synaptic plasticity. We have shown that, in rat visual cortex and in mouse PFC, HFSs of layer 2–3 (theta-burst stimulation), not only induced LTP of excitatory inputs measured from L5PyNs but also LTP of inhibitory inputs resulting in an E-I balance that remains equal to the control situation (Le Roux et al., 2006; Meunier et al., 2013). These results are in accordance with the notion of homeostatic regulation which mainly involves a dynamic adjustment of excitatory and inhibitory circuits (Turrigiano and Nelson, 2004). Because alterations in several neurotransmitter systems have been correlated with cognitive deficits resulting in impairments of learning and memory, we will focus our attention on the importance of the cooperation between different neuromodulators in the modulation of the plasticity of excitatory and inhibitory synapses. Based on our recent findings, we will highlight the importance of the coordinated action of 5-HT and DA in regulating the interaction between excitation and inhibition in the PFC, a cortical area involved in higher order cognitive functions.

## Tuning the Plasticity

### Serotonin as a Modulator

5-HT is the most widely distributed neuromodulator in the brain (Dahlström and Fuxe, 1964; Steinbusch, 1981). 5-HT is implicated in the regulation of many physiological functions such as mood, sleep, vigilance, cognitive functions, learning and memory. In the brain, serotoninergic axons originating from the raphe nucleus make synapses “en passant” and release 5-HT from varicosities (Oleskevich and Descarries, 1990) to activate a great diversity of 5-HT receptors (up to 16 types of receptors have been identified; Bockaert et al., 2006). We will focus here our attention on the role of the metabotropic 5-HT_1A_ receptor (5-HT_1A_R) which is predominant in the PFC (Santana et al., 2004).

5-HT_1A_Rs have been identified both on serotoninergic neurons (autoreceptors) where they regulate the release of 5-HT through a negative feedback and on neuronal targets of serotoninergic neurons where they function as heteroreceptors. In the cortex, the majority of postsynaptic 5-HT_1A_Rs (50%–60%) are expressed in glutamatergic neurons. In L5PyNs, 5-HT_1A_Rs are located in the soma, in the initial axonal part (Czyrak et al., 2003; Cruz et al., 2004; Santana et al., 2004) and in dendrites (Kia et al., 1996; Riad et al., 2000). We have shown for instance that in the cerebral cortex this specific distribution of 5-HTRs on pyramidal neurons is important to control output signals from the cortex (Moreau et al., 2010). In the PFC, a weaker proportion (25%) of 5-HT_1A_Rs is also expressed in GABAergic interneurons projecting onto the dendrites of pyramidal cells (Santana et al., 2004). From this distribution of 5-HT_1A_Rs in the PFC, it appears that 5-HT can regulate the excitability of both glutamatergic and GABAergic neurons (Andrade, 2011; Puig and Gulledge, 2011) but the modulatory effects of 5-HT_1A_Rs activation are not clearly understood.

Several pathologies such as mood disorders, anxiety, psychosis and fear are associated with serotonergic disorders and dysfunction of 5-HT_1A_Rs. For instance, it has been shown that the number of postsynaptic 5-HT_1A_Rs is decreased in depressive or anxious human brain (Shively et al., 2006; Lanzenberger et al., 2007; Akimova et al., 2009). One of the most commonly used pharmacological treatments of anxiety disorders is the use of selective serotonin reuptake inhibitors (SSRIs; Kasper et al., 2005). However, failures in SSRI treatment seems to be associated with the polymorphism of the gene encoding 5-HT_1A_R (Lemonde et al., 2003; Czesak et al., 2012) which might differently affect the nature (or type) of pre- vs. post-synaptic 5-HT_1A_R (Bortolozzi et al., 2012). A better understanding of the role of post-synaptic 5-HT_1A_R in the PFC is a prerequisite to the design of more selective psychoactive drugs.

### Dopamine as a Modulator

Dopaminergic fibers, mainly originating from the *Substantia nigra*, are widely distributed in all areas of the PFC and target layer 2 and layer 5 (Emson and Koob, 1978; Callier et al., 2003; Van De Werd et al., 2010). The effects of DA are mediated by the D1-class receptors (D1 and D5 receptors) and by the D2-class receptors (D2, D3 and D4 receptors; Beaulieu and Gainetdinov, 2011). Activation of the D1 class enhances the activity of adenylate cyclase whereas activation of the D2 class inhibits it (Girault and Greengard, 2004). In rat PFC, D1-like receptors (D1Rs) and D2Rs are localized on dendritic spines of L5PyNs (Gaspar et al., 1995; Negyessy and Goldman-Rakic, 2005; Paspalas and Goldman-Rakic, 2005). D2Rs are also present on GABAergic interneurons (Santana et al., 2009) while D1Rs are mainly expressed in the parvalbulmin-positive subtype of GABAergic interneurons (Glausier et al., 2009). Such a distribution of D1 and D2 receptors is again in favor of a modulatory role of DA on neuronal networks in the PFC.

Many electrophysiological studies have shown that activation of DA D1Rs favors the induction of LTP at hippocampal-PFC synapses by increasing NMDAR-mediated responses in PFC (Gurden et al., 2000; Chen et al., 2004). This enhancement of NMDA current (Shih, 2004; Tseng and O’Donnell, 2004) is explained by either the externalization of NMDA receptors (Dunah and Standaert, 2001; Dunah et al., 2004) or by their phosphorylation. D1Rs and NMDAR are co-localized in pyramidal neurons of the PFC and a direct interaction between the C-terminal domain of D1R and NR1-NR2A subunits of the NMDAR has been proposed to explain the increase in NMDA current (Kruse et al., 2009). Activation of D2Rs results in a decrease in NMDA currents (Zheng et al., 1999; Wang et al., 2003) presumably through the inhibition of the CaMKII by the PKA and the subsequent internalization of NMDARs (Wang et al., 2003). Alternatively, another signaling cascade leading to the internalization of the NR2B subunit of the NMDAR and a decrease in NMDA current has been proposed. It involves, in the PFC, an increase in the activity of the glycogen-synthase kinase-3 (GSK3) after D2R activation (Beaulieu et al., 2008; Li et al., 2009; Skinbjerg et al., 2009; Beaulieu and Gainetdinov, 2011; Sutton and Rushlow, 2011).

It is of particular interest to note that in the treatment of schizophrenia, new therapeutic strategies consist in targeting both 5-HT_1A_Rs and DA receptors by combining either 5-HT_1A_Rs agonists with D2Rs antagonists (Newman-Tancredi, 2010) or 5-HT_1A_ agonists with a D1R agonist and a D2R antagonist to prevent positive and negative symptoms of schizophrenia (Newman-Tancredi and Kleven, 2011). These clinical observations, highlight the importance of taking into account the interactions between serotoninergic and dopaminergic systems and their consequences on the physiology of the PFC, a brain area particularly associated with depressive disorders and schizophrenia.

### Complex Interactions Between Modulators of the Plasticity in the PFC

5-HT, through the activation of postsynaptic 5-HT_1A_R, is known to regulate the excitability of glutamatergic and GABAergic neurons in the PFC (Andrade, 2011; Puig and Gulledge, 2011). 5-HT modulates the induction of plasticity, depending on the 5-HT receptor subtype and brain regions (Kemp and Manahan-Vaughan, 2004). In the rat PFC, consequences of HFS on synaptic plasticity are rather complicated given that tetanic stimulations in superficial layers can induce either LTP or LTD or no plasticity. Indeed, it has been reported that such a HFS protocol can induce LTD in about one half of the cells and LTP in about one third of the cells, the remaining cells did not display any change of synaptic properties (Hirsch and Crepel, 1990, 1991; Nowicky and Bindman, 1993; Matsuda et al., 2006).

#### 5-HT_1A_ Receptors Direct the Orientation of Plasticity in Layer5 Pyramidal Neurons of the PFC

To explore the role of 5-HT_1A_Rs in the modulation of the plasticity of excitatory and inhibitory synaptic inputs to L5PyNs of the PFC, we compared the excitatory and inhibitory conductances evoked by electrical stimulations of layer 2/3 between wild-type (129/Sv WT) and 5-HT_1A_R-knock out (5-HT_1A_R-KO) mice. These transgenic mice are considered as a model for anxiety disorder (Ramboz et al., 1998) and have been designed to better understand human psychiatric conditions where a profound decrease of 5-HT_1A_R expression has been demonstrated (Sargent et al., 2000; Bhagwagar et al., 2004; Shively et al., 2006; Lanzenberger et al., 2007; Akimova et al., 2009).

**Figure 1:**
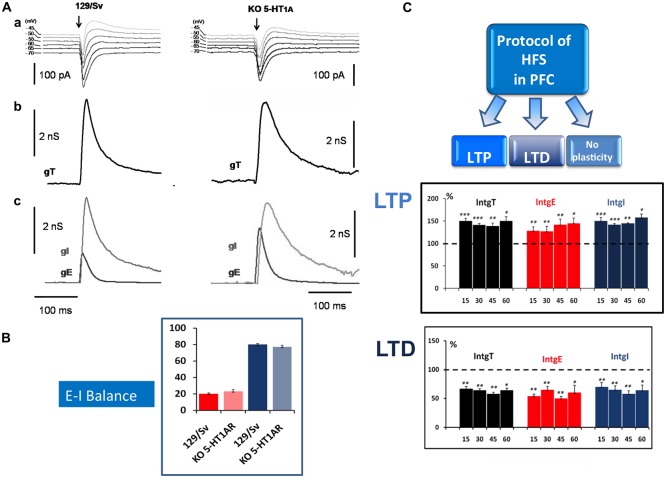
**Determination of the Excitation-Inhibition balance (E-I balance) in the prefrontal cortex (PFC) and analysis of synaptic plasticity. (A)** Brief description of the method to determine the E-I balance. **(a)** Representative current traces of synaptic responses (upper part) to layer 2–3 stimulation (arrow) recorded from a layer 5 pyramidal neuron (L5PyN) under voltage-clamp at various holding potentials in 129/Sv and KO 5-HT_1A_ mice. **(b)** The second row represents the corresponding total conductance changes (gT) at the somatic level (for a complete description of the method, see Le Roux et al., 2006; Lucas-Meunier et al., 2009). **(c)** gT was then decomposed into its Excitatory (gE) and the Inhibitory (gI) components (third row). **(B)** The percentage of excitation and inhibition is expressed as the ratio of its integral value integrals of excitatory (IntgE) and inhibitory (IntgI) to the total conductance IntgT to determine the E-I balance. We observed a significant shift of the E-I balance towards more excitation between 129/Sv mice (20%–80%) and 5-HT_1A_R-KO mice (23%–77%; Meunier et al., 2013). **(C)** High frequency stimulation (HFS) protocol (Theta burst) in layer 2–3 of the PFC induced in L5PyNs, long term potentiation (LTP) or long term depression (LTD) or no plasticity of the responses. LTP (upper insert) was recorded 15 min, 30 min, 45 min and 60 min after the stimulation. Histograms represent relative changes (compared to the control before HFS: 100%) of IntgT, IntgE, IntgI. Synaptic conductances were determined under control conditions or after HFS protocol with a low frequency of stimulation (0.05 Hz). LTD (lower insert) was recorded in the same condition. *p < 0.05, **p < 0.01, ***p < 0.001.

Our experimental approach, based on the simultaneous determination of excitatory (E) and inhibitory (I) conductance in L5PyNs, also allows the determination of the E-I balance in the PFC. Electrical stimulations of layer 2/3 (low frequency stimulation, 0.05 Hz) evoked complex postsynaptic current in L5PyNs (Figure 1A). The evoked total synaptic conductance (gT) was extracted (see Le Roux et al., 2006) and decomposed into excitatory (gE) and inhibitory (gI) conductance (Figure 1A). This allowed further evaluation of the relative contribution of evoked excitatory and inhibitory inputs reaching the soma of the recorded pyramidal neuron (Le Roux et al., 2006; Moreau et al., 2010). Calculated integrals of excitatory (IntgE) and inhibitory (IntgI) conductance were expressed as percentages of the integral of the total conductance (IntgT; Figure 1B). Analysis of recorded neurons showed that the E-I balance was significantly different in 5-HT_1A_R-KO mutants (23%–77%) compared to 129/Sv WT mice (20%–80%; Figure 1B). In control 129/Sv WT mice and in the presence of WAY 100635 (a 5-HT_1A_R selective antagonist) the E-I balance was shifted towards more excitation (24%–76%), a value similar to what we observed in 5HT_1A_R-KO mice (Meunier et al., 2013). These results show that L5PyNs in 129/Sv mice express functional 5HT_1A_Rs and that 5HT_1A_Rs play a crucial role in tuning the E-I balance in L5PyNs of the PFC.

**Figure 2:**
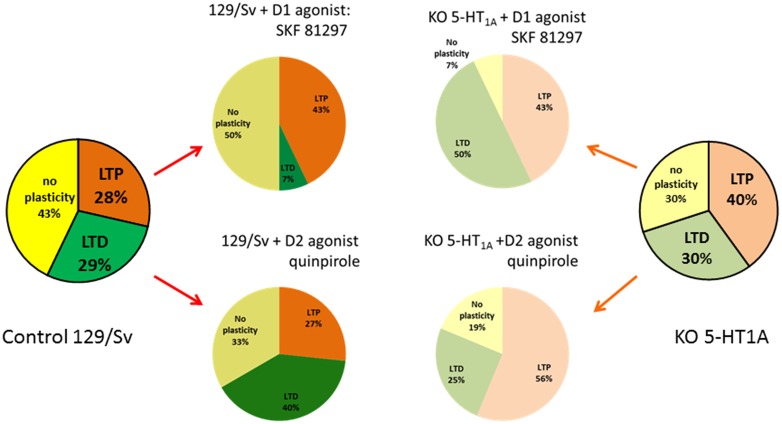
**Schematic summary of the orientation of the plasticity in L5PyNs of the PFC.** Ratio of L5PyNs displaying LTP, LTD or no plasticity calculated 45 min after HFS under different conditions. In each case, the analysis of the neuronal population results from 15 independent experiments. Left and right charts were obtained from control 129/Sv mice or from 5-HT_1A_R-knock out (5-HT_1A_R-KO) mice. Note that the ratio of L5PyNs displaying LTP was significantly increased in 5-HT_1A_R-KO mice (see Meunier et al., 2013). In 129/Sv mice, the D1-like receptors (D1Rs) agonist, SKF 81297, markedly reduced the proportion of L5PyNs displaying LTD while it enhanced the ratio of L5PyNs displaying LTP (Meunier et al., 2015). The D2Rs agonist, quinpirole, markedly increased the ratio of L5PyNs displaying LTD (from Meunier et al., 2017). In 5-HT_1A_R-KO mice, the D1Rs agonist, SKF 81297, markedly increased the proportion of L5PyNs displaying LTD while it reduced the ratio of L5PyNs displaying LTP. Activation of D2R with quinpirole had no significant effect on the ratio of L5PyNs displaying LTD.

We observed that, in both strains, HFS (theta burst) induced either LTP or LTD of both excitatory and inhibitory conductance or no plasticity (Figure 1C). These changes in synaptic efficacy were due to the activation of NMDARs since their blockade with D-L-AP5 prevented the induction of synaptic plasticity. When comparing 129/Sv WT and 5HT_1A_R-KO mice showing LTP of excitatory and inhibitory synaptic transmission, we observed that the absence of functional 5HT_1A_R resulted in a reduced potentiation of excitatory synaptic transmission while the potentiation of inhibition remained unaffected (Meunier et al., 2013). The use of a specific 5-HT_1A_R antagonist confirmed the role of 5-HT_1A_Rs in the modulation of LTP of excitation in the PFC. We also observed that the proportion (calculated from the whole neuronal population) of L5PyNs displaying either LTP or LTD or no change were different between 129/Sv WT and 5-HT_1A_R-KO mice. In 5-HT_1A_R-KO mice, the percentage of neurons displaying LTP was significantly increased compared to 129/Sv WT mice (Figure 2) whereas the percentage of neurons showing LTD remained identical. Our results indicate that 5-HT_1A_Rs play an important role in the orientation of the synaptic plasticity of L5PyNs in the PFC towards either LTP or LTD or no plasticity (Meunier et al., 2013).

It is established that different forms of synaptic plasticity such as LTP and LTD are induced by NMDAR activation (Stanton, 1996) that leads to the control of the trafficking of AMPA receptors. The externalization (Malenka and Nicoll, 1999; Malinow and Malenka, 2002) or the internalization (Beattie et al., 2000; Hanley and Henley, 2005; Fernández-Monreal et al., 2012) of AMPA receptors are commonly considered to be responsible for LTP and LTD induction respectively. The orientation of the synaptic plasticity towards LTP or LTD relying on AMPARs trafficking is known to be correlated with the magnitude of the dendritic calcium signal (Cormier et al., 2001). We observed that the activation of 5-HT_1A_Rs enhanced evoked NMDA currents, indicating that 5-HT_1A_Rs modulate NMDARs (Meunier et al., 2013). Therefore we propose that the increase in the percentage of neurons displaying LTP observed in the absence of functional 5-HT_1A_Rs (5-HT_1A_R-KO mice) could result from a reduced calcium influx through NMDARs. So it appears that, in the PFC, 5-HT_1A_Rs limit the induction of LTP and favor LTD by modulating the NMDA currents.

#### D1R-Mediated Modulation of Synaptic Plasticity Depends on Functional 5HT_1A_R

At hippocampal-PFC synapses, DA (acting at D1Rs) is known to favor LTP induction (Gurden et al., 2000; Chen et al., 2004). We have shown recently (Meunier et al., 2015) that a high frequency protocol of stimulation (HFS) in the presence of the D1R agonist SKF 81297 results in an increase of the population of L5PyNs displaying LTP in 129/Sv mice (Figure 2). By contrast, we observed an increase in the amount of neurons showing LTD when D1R were activated in the absence of functional 5-HT_1A_Rs (KO). This cooperation between 5-HT_1A_Rs and D1Rs to determine the direction of the synaptic plasticity was confirmed by a pharmacological approach (Meunier et al., 2015). We also observed in 129/Sv mice, an increase in the number of L5PyNs in the “no plasticity” class after D1Rs activation (Figure 2).

This orientation of the plasticity could be the result of a modulation of NMDA-mediated currents by D1Rs and 5-HT_1A_Rs. A strong increase in intracellular calcium concentration within the dendritic spines of L5PyNs would facilitate LTP induction whereas a weaker calcium signal would facilitate LTD induction. D1Rs activation is known to modulate NMDARs and this modulation relies on the expression of different NMDARs subunits (NR2B vs. NR2A; Liu et al., 2006; Varela et al., 2009). The subunit composition of NMDARs determines the amplitude and the duration of the transient calcium signal that will lead direct synaptic plasticity towards either LTP or LTD (Mulkey and Malenka, 1992; Huang et al., 2010). We therefore make the assumption that the cooperation between 5-HT_1A_Rs and D1Rs would set the magnitude of the calcium influx through NMDARs (Meunier et al., 2015). The resulting calcium signal at the dendrite would lead to the modulation of the CamKII activity (Chen et al., 2007; Ashpole et al., 2012; Coultrap et al., 2014) to control the trafficking of AMPARs at the synapse and consequently determine the direction of the synaptic plasticity. When D1Rs are activated in the absence of functional 5-HT_1A_Rs (5-HT_1A_R-KO mice), the increased proportion of neurons showing LTD responses (Meunier et al., 2015) could then be the result of reduced NMDA currents due to changes in NMDARs subunits composition.

It cannot be excluded that other signaling pathways following 5-HT_1A_Rs and D1Rs activation play a role in AMPARs trafficking and in the modulation of synaptic plasticity. Following 5-HT_1A_R activation (Polter and Li, 2010) as well as D1R activation (Gurden et al., 2000; Huang et al., 2004; Kruse et al., 2009), the cAMP/PKA signaling cascade could also be involved in regulating the incorporation of AMPARs at the synapse.

There is also growing evidence that D1/D5R stimulation leads to Gq-dependent activation of PLC, IP3-mediated Ca^2+^ release and CamKII activation (Chen et al., 2007). It is important to note that several studies have reported that metabotropic receptor activation coupled to PLC stimulation lead to LTD induction (Choi et al., 2005). In the 5-HT_1A_Rs-KO mice, the D1Rs effects could mostly rely on the regulation of PLC activity.

#### Activation of D2R in the Presence of Functional 5-HT_1A_Rs Promotes HFS-Induced LTD of Excitatory Synapses via the Activation of GSK3

DA is known to modulate NMDAR- and AMPAR-mediated currents, neuronal excitability and synaptic plasticity towards LTD through the activation of D2Rs. Both DA and 5-HT can regulate neuronal activity via a common signaling pathway involving the regulation of the activity of GSK3, a serine/threonine kinase playing an important role in the regulation of several receptors for neurotransmitters (Chen et al., 2009).

Two isoforms of GSK3 have been identified (GSK3α and GSK3β; Woodgett, 1990, 2001) which can be inactivated through their phosphorylation by Akt (Cross et al., 1995; Frame et al., 2001). It has been reported that, in the PFC and the striatum, the activation of D2Rs induces the activation of GSK3 (Beaulieu et al., 2004, 2008; Chen et al., 2009; Beaulieu and Gainetdinov, 2011). This activation of GSK3β has been shown to promote the internalization of the NR2B subunit of NMDARs leading in the rat hippocampus to the induction of LTD (Peineau et al., 2007) while LTP could be favored by higher levels of phosphorylation of GSK3β (Hooper et al., 2007; Peineau et al., 2007). It has also been observed that GSK3 can modulate NMDARs and AMPARs through either 5-HT_1A_R or 5-HT_2A_R activation (Li et al., 2004, 2007).

Importantly, like 5-HT_1A_Rs and D2Rs, GSK3 is also a molecular target in the treatment of neuropsychiatric disorders such as major depression and anxiety (Meltzer et al., 2003; Meltzer and Massey, 2011). We therefore investigated the possible cooperation between 5-HT_1A_R and D2R in the modulation of synaptic plasticity within the PFC. We have shown that the activation of D2Rs and the downstream activation of GSK3 favor the induction of LTD of excitatory synaptic transmission (Meunier et al., 2017). In addition, we demonstrated that this control of synaptic plasticity via GSK3 requires the presence of functional 5-HT_1A_Rs. We therefore made the hypothesis of cooperation between 5-HT_1A_R and D2R, via the regulation of GSK3, to modulate excitatory synaptic transmission. This hypothesis is supported by recent observations showing that D2R and 5-HT_1A_R can modulate the phosphorylation or the dephosphorylation of GSK3β (the dephosphorylated form being the active form).

The pathway downstream D2R activation leads, via the regulation of cyclic AMP production and β-arrestin activity, to the dephosphorylation (deactivation) of Akt and consequently to the dephosphorylation (activation) of GSK3 by the protein phosphatase 2A (PP2A Beaulieu et al., 2004, 2005, 2008, 2009; Beurel et al., 2015). There are apparent contradictory observations regarding the regulation of GSK3β by 5-HT receptors. It has been reported, in rat hippocampus, that the activation of 5-HT_1A_Rs increases the phosphorylation of GSK3β by Akt, promoting the inactivated form (Polter et al., 2012). Others have suggested that 5-HT_1A_Rs activation could activate GSK3 via the phosphorylation of Akt by PP2A (Hsiung et al., 2008). We have shown, in the PFC of 5-HT_1A_R-KO mice, an increase in the phosphorylated form of GSK3 (Meunier et al., 2017). This suggests that in the PFC, the activation of 5-HT_1A_Rs promotes the active form of GSK3 and leads to the internalization of AMPARs and to the induction of LTD of excitatory synapses.

Our results highlight the importance of the cooperation between D2Rs and 5-HT_1A_Rs in the regulation of the E-I balance and of the plasticity of excitatory synapses in the PFC. 5-HT_2A_R activation is also known to have a clear excitatory effect on L5PyNs and layer 2–3 GABAergic interneurons in the PFC (Andrade, 2011; Celada et al., 2013) to modulate NMDARs and AMPARs through GSK3 (Li et al., 2004, 2007). It it thus very likely that 5-HT_2A_R activation plays a role in the modulation of the E-I balance and in the modulation of long-term synaptic plasticity by DA. The functional dimerization between D2R and 5-HT_2A_R (Franklin and Carrasco, 2012) that can be modulated by cannabinoid receptors, introduces another level of complexity that has to be taken in account. Recent findings indicate a prominent co-localization of D2Rs and 5-HT_1A_Rs in the neurons of the PFC compared to other brain regions (Łukasiewicz et al., 2016) suggesting that D2Rs-5-HT_1A_Rs heteromers may be expressed in the PFC. When expressed in HEK cells (Łukasiewicz et al., 2016), it appears that the activation of such heteromers recruits metabolic pathways different from those downstream the activation of homomeric 5-HT_1A_ or D2 receptors. This could open up new therapeutic strategies based on the selective activation of one or the other metabolic pathways to improve the treatment of pathologies such as schizophrenia.

#### Schematic Overview and Perspectives

**Figure 3:**
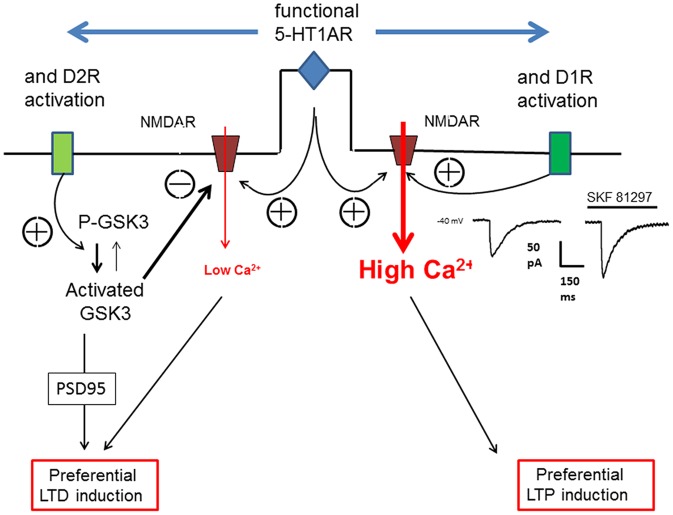
**Schematic view of the action of 5-HT_1A_R on D1R or D2R to orientate the plasticity of an excitatory synapse in the PFC.** The orientation of synaptic plasticity by dopamine (DA) acting at either D1Rs or D2Rs is dependent on the concomitant activation of 5-HT_1A_Rs. We assume that the induction of either LTP or LTD of excitatory synapses depends on the magnitude of calcium transient (controlling AMPARs trafficking) within the dendritic spines which is determined by the calcium influx through NMDARs. Therefore, an increased calcium transient (High Ca^2+^) following 5-HT_1A_R and D1R activation (see the NMDA current on the right inset) would lead to LTP whereas a reduced calcium transient (low Ca^2+^) following 5-HT_1A_R and D2R activation would lead, via the activation of glycogen-synthase kinase-3 (GSK3), to LTD.

Based on our own research, we focused here our attention on the role of 5-HT_1A_Rs in the dopaminergic modulation of long-term synaptic plasticity. The modulatory effect of D1 or D2R on excitatory synaptic plasticity depends on the presence of functional 5-HT_1A_Rs. It appears that 5-HT_1A_R cooperate with either D1R or D2R to direct the long term plasticity towards either LTP (D1Rs) or LTD (D2R). We assume that it is the cooperation of 5-HT_1A_Rs with either D1Rs or D2Rs that will determine the magnitude of the Ca^2+^ influx through NMDARs evoked by high frequency synaptic stimulation and consequently favor either LTP or LTD. As illustrated in Figure 3, we consider the Ca^2+^ transient within dendritic spines as a key element of long-term synaptic plasticity. However it remains to determine how the recruitment of different signaling pathways following the activation of D1Rs, D2Rs and 5-HT_1A_Rs leads to the regulation of AMPAR trafficking at the synapse which determines the synaptic strength. The cooperation between 5-HT and DA receptors in the modulation of the long-term plasticity of excitatory synaptic inputs may have consequences on neuronal output (spiking behavior) and information processing within the PFC. The absence of modulatory effects on inhibitory synaptic inputs would result in imbalanced changes in synaptic strength in favor of excitation affecting the threshold of neuronal input-output functions (Carvalho and Buonomano, 2009).

GSK3β is considered as a key player in synaptic plasticity (reviewed in Bradley et al., 2012). It is therefore important to understand the factors regulating GSK3β activity in the perspective of adapting therapy for several neurological and psychiatric disorders in which a dysregulation of GSK3β activity has been reported (Beurel et al., 2015). Mood disorders (major depressive disorder and bipolar disorder) are characterized by a disruption of the fine equilibrium between 5-HT and DA systems regulating the balance between the inactive and the active form of GSK3β at the synaptic level. Many therapeutic strategies using Li^+^ as a blocker of GSK3ß activity have been considered as an attempt to resettle such a balance (Jope, 2011). Our results suggest that in the case of pathologies such as major depression for which a reduction of the number of 5-HT_1A_Rs is observed, new therapeutic strategies resetting the equilibrium between the active and the inactive form of GSK3ß could rather consist in acting at both 5-HT and DA receptors.

## Author Contributions

CNJM designed and conducted the experiments, wrote the article. PC wrote the article. PMF designed the experiments and wrote the article.

## Funding

Centre National de la Recherche Scientifique (CNRS) is our institution which has provided basic funding.

## Conflict of Interest Statement

The authors declare that the research was conducted in the absence of any commercial or financial relationships that could be construed as a potential conflict of interest.

## References

[B1] Abeles M. (1991). Corticonomics: Neuronal Circuits of the Cerebral Cortex. Cambridge, MA: Cambridge University Press.

[B2] Adermark L. Lovinger D. M. (2009). Frequency-dependent inversion of net striatal output by endocannabinoid-dependent plasticity at different synaptic inputs. J. Neurosci. 29, 1375–1380. 10.1523/JNEUROSCI.3842-08.2009 19193884 PMC2744205

[B3] Akimova E. Lanzenberger R. Kasper S. (2009). The serotonin-1A receptor in anxiety disorders. Biol. Psychiatry 66, 627–635. 10.1016/j.biopsych.2009.03.012 19423077

[B4] Anderson J. Lampl I. Reichova I. Carandini M. Ferster D. (2000). Stimulus dependence of two-state fluctuations of membrane potential in cat visual cortex. Nat. Neurosci. 3, 617–621. 10.1038/75797 10816319

[B5] Andrade R. (2011). Serotonergic regulation of neuronal excitability in the prefrontal cortex. Neuropharmacology 61, 382–386. 10.1016/j.neuropharm.2011.01.015 21251917 PMC3110517

[B6] Arancibia-Cárcamo I. L. Kittler J. T. (2009). Regulation of GABA_A_ receptor membrane trafficking and synaptic localization. Pharmacol. Ther. 123, 17–31. 10.1016/j.pharmthera.2009.03.012 19374920

[B7] Ashpole N. M. Song W. Brustovetsky T. Engleman E. A. Brustovetsky N. Cummins T. R.. (2012). Calcium/calmodulin-dependent protein kinase II (CaMKII) inhibition induces neurotoxicity via dysregulation of glutamate/calcium signaling and hyperexcitability. J. Biol. Chem. 287, 8495–8506. 10.1074/jbc.m111.323915 22253441 PMC3318689

[B8] Azad S. C. Monory K. Marsicano G. Cravatt B. F. Lutz B. Zieglgänsberger W.. (2004). Circuitry for associative plasticity in the amygdala involves endocannabinoid signaling. J. Neurosci. 24, 9953–9961. 10.1523/JNEUROSCI.2134-04.2004 15525780 PMC6730232

[B9] Bear M. F. Malenka R. C. (1994). Synaptic plasticity: LTP and LTD. Curr. Opin. Neurobiol. 4, 389–399. 10.1016/0959-4388(94)90101-5 7919934

[B10] Beattie E. C. Carroll R. C. Yu X. Morishita W. Yasuda H. von Zastrow M.. (2000). Regulation of AMPA receptor endocytosis by a signaling mechanism shared with LTD. Nat. Neurosci. 3, 1291–1300. 10.1038/81823 11100150

[B12] Beaulieu J. Gainetdinov R. R. (2011). The physiology, signaling, and pharmacology of dopamine receptors. Pharmacol. Rev. 63, 182–217. 10.1124/pr.110.002642 21303898

[B13] Beaulieu J. M. Gainetdinov R. R. Caron M. G. (2009). Akt/GSK3 signaling in the action of psychotropic drugs. Annu. Rev. Pharmacol. Toxicol. 49, 327–347. 10.1146/annurev.pharmtox.011008.145634 18928402

[B300] Beaulieu J. M. Sotnikova T. D. Marion S. Lefkowitz R. J. Gainetdinov R. R. Caron M. G. (2005). An Akt/β-arrestin 2/PP2A signaling complex mediates dopaminergic neurotransmission and behavior. Cell 122, 261–273. 10.1016/j.cell.2005.05.012 16051150

[B14] Beaulieu J. M. Sotnikova T. D. Yao W. D. Kockeritz L. Woodgett J. R. Gainetdinov R. R.. (2004). Lithium antagonizes dopamine-dependent behaviors mediated by an AKT/glycogen synthase kinase 3 signaling cascade. Proc. Natl. Acad. Sci. U S A 101, 5099–5104. 10.1073/pnas.0307921101 15044694 PMC387380

[B11] Beaulieu J.-M. Zhang X. Rodriguiz R. M. Sotnikova T. D. Cools M. J. Wetsel W. C.. (2008). Role of GSK3β in behavioral abnormalities induced by serotonin deficiency. Proc. Natl. Acad. Sci. U S A 105, 1333–1338. 10.1073/pnas.0711496105 18212115 PMC2234138

[B15] Berger T. K. Silberberg G. Perin R. Markram H. (2010). Brief bursts self-inhibit and correlate the pyramidal network. PLoS Biol. 8:e1000473. 10.1371/journal.pbio.1000473 20838653 PMC2935452

[B16] Beurel E. Grieco S. F. Jope R. S. (2015). Glycogen synthase kinase-3 (GSK3): regulation, actions, and diseases. Pharmacol. Ther. 148, 114–131. 10.1016/j.pharmthera.2014.11.016 25435019 PMC4340754

[B17] Bhagwagar Z. Rabiner E. A. Sargent P. A. Grasby P. M. Cowen P. J. (2004). Persistent reduction in brain serotonin1A receptor binding in recovered depressed men measured by positron emission tomography with [^11^C]WAY-100635. Mol. Psychiatry 9, 386–392. 10.1038/sj.mp.4001401 15042104

[B18] Bliss T. V. Lomo T. (1973). Long-lasting potentiation of synaptic transmission in the dentate area of the anaesthetized rabbit following stimulation of the perforant path. J. Physiol. 232, 331–356. 10.1113/jphysiol.1973.sp010273 4727084 PMC1350458

[B19] Bockaert J. Claeysen S. Bécamel C. Dumuis A. Marin P. (2006). Neuronal 5-HT metabotropic receptors: fine-tuning of their structure, signaling, and roles in synaptic modulation. Cell Tissue Res. 326, 553–572. 10.1007/s00441-006-0286-1 16896947

[B56] den Boon F. S. Werkman T. R. Schaafsma-Zhao Q. Houthuijs K. Vitalis T. Kruse C. G.. (2015). Activation of type-1 cannabinoid receptor shifts the balance between excitation and inhibition towards excitation in layer II/III pyramidal neurons of the rat prelimbic cortex. Pflugers Arch. 467, 1551–1564. 10.1007/s00424-014-1586-z 25081244

[B20] Borah A. Mohanakumar K. P. (2007). Long-term L-DOPA treatment causes indiscriminate increase in dopamine levels at the cost of serotonin synthesis in discrete brain regions of rats. Cell. Mol. Neurobiol. 27, 985–996. 10.1007/s10571-007-9213-6 17934805 PMC11517132

[B21] Borg-Graham L. J. Monier C. Frégnac Y. (1998). Visual input evokes transient and strong shunting inhibition in visual cortical neurons. Nature 393, 369–373. 10.1038/30735 9620800

[B22] Bortolozzi A. Castañé A. Semakova J. Santana N. Alvarado G. Cortés R.. (2012). Selective siRNA-mediated suppression of 5-HT_1A_ autoreceptors evokes strong anti-depressant-like effects. Mol. Psychiatry 17, 612–623. 10.1038/mp.2011.92 21808255

[B23] Bradley C. A. Peineau S. Taghibiglou C. Nicolas C. S. Whitcomb D. J. Bortolotto Z. A.. (2012). A pivotal role of GSK-3 in synaptic plasticity. Front. Mol. Neurosci. 5:13. 10.3389/fnmol.2012.00013 22363262 PMC3279748

[B24] Burnashev N. Schoepfer R. Monyer H. Ruppersberg J. P. Günther W. Seeburg P. H.. (1992). Control by asparagine residues of calcium permeability and magnesium blockade in the NMDA receptor. Science 257, 1415–1419. 10.1126/science.1382314 1382314

[B25] Calabresi P. Pisani A. Mercuri N. B. Bernardi G. (1992). Long-term potentiation in the striatum is unmasked by removing the voltage-dependent magnesium block of NMDA receptor channels. Eur. J. Neurosci. 4, 929–935. 10.1111/j.1460-9568.1992.tb00119.x 12106428

[B26] Callier S. Snapyan M. Le Crom S. Prou D. Vincent J.-D. Vernier P. (2003). Evolution and cell biology of dopamine receptors in vertebrates. Biol. Cell 95, 489–502. 10.1016/s0248-4900(03)00089-3 14597267

[B27] Camiré O. Topolnik L. (2012). Functional compartmentalisation and regulation of postsynaptic Ca^2+^ transients in inhibitory interneurons. Cell Calcium 52, 339–346. 10.1016/j.ceca.2012.05.001 22656961

[B28] Carta M. Carlsson T. Kirik D. Björklund A. (2007). Dopamine released from 5-HT terminals is the cause of L-DOPA-induced dyskinesia in parkinsonian rats. Brain 130, 1819–1833. 10.1093/brain/awm082 17452372

[B29] Carvalho T. P. Buonomano D. V. (2009). Differential effects of excitatory and inhibitory plasticity on synaptically driven neuronal input-output functions. Neuron 61, 774–785. 10.1016/j.neuron.2009.01.013 19285473 PMC2676350

[B30] Castillo P. E. Chiu C. Q. Carroll R. C. (2011). Long-term plasticity at inhibitory synapses. Curr. Opin. Neurobiol. 21, 328–338. 10.1016/j.conb.2011.01.006 21334194 PMC3092861

[B31] Celada P. Puig M. V. Artigas F. (2013). Serotonin modulation of cortical neurons and networks. Front. Integr. Neurosci. 7:25. 10.3389/fnint.2013.00025 23626526 PMC3630391

[B33] Chen L. Bohanick J. D. Nishihara M. Seamans J. K. Yang C. R. (2007). Dopamine D1/5 receptor-mediated long-term potentiation of intrinsic excitability in rat prefrontal cortical neurons: Ca^2+^-dependent intracellular signaling. J. Neurophysiol. 97, 2448–2464. 10.1152/jn.00317.2006 17229830

[B32] Chen G. Greengard P. Yan Z. (2004). Potentiation of NMDA receptor currents by dopamine D1 receptors in prefrontal cortex. Proc. Natl. Acad. Sci. U S A 101, 2596–2600. 10.1073/pnas.0308618100 14983054 PMC356995

[B34] Chen L. Salinas G. D. Li X. (2009). Regulation of serotonin 1B receptor by glycogen synthase kinase-3. Mol. Pharmacol. 76, 1150–1161. 10.1124/mol.109.056994 19741007 PMC2784733

[B35] Chevaleyre V. Castillo P. E. (2003). Heterosynaptic LTD of hippocampal GABAergic synapses: a novel role of endocannabinoids in regulating excitability. Neuron 38, 461–472. 10.1016/S0896-6273(03)00235-6 12741992

[B36] Chevaleyre V. Takahashi K. A. Castillo P. E. (2006). Endocannabinoid-mediated synaptic plasticity in the CNS. Annu. Rev. Neurosci. 29, 37–76. 10.1146/annurev.neuro.29.051605.112834 16776579

[B37] Choi S. Y. Chang J. Jiang B. Seol G. H. Min S. S. Han J. S.. (2005). Multiple receptors coupled to phospholipase C gate long-term depression in visual cortex. J. Neurosci. 25, 11433–11443. 10.1523/JNEUROSCI.4084-05.2005 16339037 PMC6725895

[B38] Cobos I. Calcagnotto M. E. Vilaythong A. J. Thwin M. T. Noebels J. L. Baraban S. C.. (2005). Mice lacking Dlx1 show subtype-specific loss of interneurons, reduced inhibition and epilepsy. Nat. Neurosci. 8, 1059–1068. 10.1038/nn1499 16007083

[B39] Compte A. Sanchez-Vives M. V. McCormick D. A. Wang X. J. (2003). Cellular and network mechanisms of slow oscillatory activity (<1 Hz) and wave propagations in a cortical network model. J. Neurophysiol. 89, 2707–2725. 10.1152/jn.00845.2002 12612051

[B40] Contreras D. Timofeev I. Steriade M. (1996). Mechanisms of long-lasting hyperpolarizations underlying slow sleep oscillations in cat corticothalamic networks. J. Physiol. 494, 251–264. 10.1113/jphysiol.1996.sp021488 8814619 PMC1160627

[B41] Cormier R. J. Greenwood A. C. Connor J. A. (2001). Bidirectional synaptic plasticity correlated with the magnitude of dendritic calcium transients above a threshold. J. Neurophysiol. 85, 399–406. 11152740 10.1152/jn.2001.85.1.399

[B42] Cossart R. Bernard C. Ben-Ari Y. (2005). Multiple facets of GABAergic neurons and synapses: multiple fates of GABA signalling in epilepsies. Trends Neurosci. 28, 108–115. 10.1016/j.tins.2004.11.011 15667934

[B43] Cossart R. Dinocourt C. Hirsch J. C. Merchan-Perez A. De Felipe J. Ben-Ari Y.. (2001). Dendritic but not somatic GABAergic inhibition is decreased in experimental epilepsy. Nat. Neurosci. 4, 52–62. 10.1038/82900 11135645

[B44] Costa C. Tozzi A. Siliquini S. Galletti F. Cardaioli G. Tantucci M.. (2011). A critical role of NO/cGMP/PKG dependent pathway in hippocampal post-ischemic LTP: modulation by zonisamide. Neurobiol. Dis. 44, 185–191. 10.1016/j.nbd.2011.06.015 21749921

[B45] Coultrap S. J. Freund R. K. O’Leary H. Sanderson J. L. Roche K. W. Dell’Acqua M. L.. (2014). Autonomous CaMKII mediates both LTP and LTD using a mechanism for differential substrate site selection. Cell Rep. 6, 431–437. 10.1016/j.celrep.2014.01.005 24485660 PMC3930569

[B46] Coyle J. T. (2006). Substance use disorders and Schizophrenia: a question of shared glutamatergic mechanisms. Neurotox. Res. 10, 221–233. 10.1007/bf03033359 17197372

[B47] Creese I. Burt D. R. Snyder S. H. (1976). Dopamine receptor binding predicts clinical and pharmacological potencies of antischizophrenic drugs. Science 192, 481–483. 10.1126/science.3854 3854

[B48] Cross D. A. Alessi D. R. Cohen P. Andjelkovich M. Hemmings B. A. (1995). Inhibition of glycogen synthase kinase-3 by insulin mediated by protein kinase B. Nature 378, 785–789. 10.1038/378785a0 8524413

[B49] Cruz D. A. Eggan S. M. Azmitia E. C. Lewis D. A. (2004). Serotonin1A receptors at the axon initial segment of prefrontal pyramidal neurons in schizophrenia. Am. J. Psychiatry 161, 739–742. 10.1176/appi.ajp.161.4.739 15056522

[B50] Czarnecki A. Birtoli B. Ulrich D. (2007). Cellular mechanisms of burst firing-mediated long-term depression in rat neocortical pyramidal cells. J. Physiol. 578, 471–479. 10.1113/jphysiol.2006.123588 17082228 PMC2075152

[B51] Czesak M. Le François B. Millar A. M. Deria M. Daigle M. Visvader J. E.. (2012). Increased serotonin-1A (5-HT1A) autoreceptor expression and reduced raphe serotonin levels in deformed epidermal autoregulatory factor-1 (Deaf-1) gene knock-out mice. J. Biol. Chem. 287, 6615–6627. 10.1074/jbc.m111.293027 22232550 PMC3307310

[B52] Czyrak A. Czepiel K. Mackowiak M. Chocyk A. Wedzony K. (2003). Serotonin 5-HT1A receptors might control the output of cortical glutamatergic neurons in rat cingulate cortex. Brain Res. 989, 42–51. 10.1016/s0006-8993(03)03352-3 14519510

[B53] Dahlström A. Fuxe K. (1964). Localization of monoamines in the lower brain stem. Experientia 20, 398–399. 10.1007/bf02147990 5856530

[B54] Dani V. S. Chang Q. Maffei A. Turrigiano G. G. Jaenisch R. Nelson S. B. (2005). Reduced cortical activity due to a shift in the balance between excitation and inhibition in a mouse model of Rett syndrome. Proc. Natl. Acad. Sci. U S A 102, 12560–12565. 10.1073/pnas.0506071102 16116096 PMC1194957

[B55] Daoudal G. Debanne D. (2003). Long-term plasticity of intrinsic excitability: learning rules and mechanisms. Learn. Mem. 10, 456–465. 10.1101/lm.64103 14657257

[B57] Dudek S. M. Bear M. F. (1992). Homosynaptic long-term depression in area CA1 of hippocampus and effects of N-methyl-D-aspartate receptor blockade. Proc. Natl. Acad. Sci. U S A 89, 4363–4367. 10.1073/pnas.89.10.4363 1350090 PMC49082

[B58] Dunah A. W. Sirianni A. C. Fienberg A. A. Bastia E. Schwarzschild M. A. Standaert D. G. (2004). Dopamine D1-dependent trafficking of striatal N-methyl-D-aspartate glutamate receptors requires Fyn protein tyrosine kinase but not DARPP-32. Mol. Pharmacol. 65, 121–129. 10.1124/mol.65.1.121 14722243

[B59] Dunah A. W. Standaert D. G. (2001). Dopamine D1 receptor-dependent trafficking of striatal NMDA glutamate receptors to the postsynaptic membrane. J. Neurosci. 21, 5546–5558. 11466426 10.1523/JNEUROSCI.21-15-05546.2001 PMC6762635

[B60] Egorov A. V. Hamam B. N. Fransén E. Hasselmo M. E. Alonso A. A. (2002). Graded persistent activity in entorhinal cortex neurons. Nature 420, 173–178. 10.1038/nature01171 12432392

[B61] Emson P. C. Koob G. F. (1978). The origin and distribution of dopamine-containing afferents to the rat frontal cortex. Brain Res. 142, 249–267. 10.1016/0006-8993(78)90634-0 24492

[B62] Etkin A. Alarcón J. M. Weisberg S. P. Touzani K. Huang Y. Y. Nordheim A.. (2006). A role in learning for SRF: deletion in the adult forebrain disrupts LTD and the formation of an immediate memory of a novel context. Neuron 50, 127–143. 10.1016/j.neuron.2006.03.013 16600861

[B63] Feldman D. E. (2009). Synaptic mechanisms for plasticity in neocortex. Annu. Rev. Neurosci. 32, 33–55. 10.1146/annurev.neuro.051508.135516 19400721 PMC3071739

[B64] Fernández-Monreal M. Brown T. C. Royo M. Esteban J. A. (2012). The balance between receptor recycling and trafficking toward lysosomes determines synaptic strength during long-term depression. J. Neurosci. 32, 13200–13205. 10.1523/JNEUROSCI.0061-12.2012 22993436 PMC6621469

[B65] Frame S. Cohen P. Biondi R. M. (2001). A common phosphate binding site explains the unique substrate specificity of GSK3 and its inactivation by phosphorylation. Mol. Cell 7, 1321–1327. 10.1016/s1097-2765(01)00253-2 11430833

[B66] Franklin J. M. Carrasco G. A. (2012). Cannabinoid-induced enhanced interaction and protein levels of serotonin 5-HT2A and dopamine D2 receptors in rat prefrontal cortex. J. Psychopharmacol. 26, 1333–1347. 10.1177/0269881112450786 22791651 PMC3746962

[B67] Fuster J. M. (2001). The prefrontal cortex—an update: time is of the essence. Neuron 30, 319–333. 10.1016/S0896-6273(01)00285-9 11394996

[B68] Gaiarsa J. L. Ben-Ari J. (2006). “Long-term plasticity at inhibitory synapses: a phenomenon that has been overlooked,” in The Dynamic Synapse: Molecular Methods in Ionotropic Receptor Biology, eds Kittler J. T. Moss S. J. (Boca Raton, FL: CRC Press), Chapter 2, 23–36.

[B69] Gaspar P. Bloch B. Le Moine C. (1995). D1 and D2 receptor gene expression in the rat frontal cortex: cellular localization in different classes of efferent neurons. Eur. J. Neurosci. 7, 1050–1063. 10.1111/j.1460-9568.1995.tb01092.x 7613610

[B70] Girault J.-A. Greengard P. (2004). The neurobiology of dopamine signaling. Arch. Neurol. 61, 641–644. 10.1001/archneur.61.5.641 15148138

[B71] Glausier J. R. Khan Z. U. Muly E. C. (2009). Dopamine D1 and D5 receptors are localized to discrete populations of interneurons in primate prefrontal cortex. Cereb. Cortex 19, 1820–1834. 10.1093/cercor/bhn212 19020206 PMC2705695

[B72] Goldberg J. H. Yuste R. Tamas G. (2003). Ca^2+^ imaging of mouse neocortical interneurone dendrites: contribution of Ca^2+^-permeable AMPA and NMDA receptors to subthreshold Ca^2+^dynamics. J. Physiol. 551, 67–78. 10.1113/jphysiol.2003.042598 12844507 PMC2343145

[B73] Grover L. M. Teyler T. J. (1994). Activation of NMDA receptors in hippocampal area CA1 by low and high frequency orthodromic stimulation and their contribution to induction of long-term potentiation. Synapse 16, 66–75. 10.1002/syn.890160108 7907824

[B74] Gubellini P. Ben-Ari Y. Gaïarsa J.-L. (2005). Endogenous neurotrophins are required for the induction of GABAergic long-term potentiation in the neonatal rat hippocampus. J. Neurosci. 25, 5796–5802. 10.1523/JNEUROSCI.0824-05.2005 15958746 PMC6724877

[B75] Gurden H. Takita M. Jay T. M. (2000). Essential role of D1 but not D2 receptors in the NMDA receptor-dependent long-term potentiation at hippocampal-prefrontal cortex synapses *in vivo*. J. Neurosci. 20:RC106. 11069975 10.1523/JNEUROSCI.20-22-j0003.2000 PMC6773154

[B76] Haider B. Duque A. Hasenstaub A. R. McCormick D. A. (2006). Neocortical network activity *in vivo* is generated through a dynamic balance of excitation and inhibition. J. Neurosci. 26, 4535–4545. 10.1523/JNEUROSCI.5297-05.2006 16641233 PMC6674060

[B77] Haider B. McCormick D. A. (2009). Rapid neocortical dynamics: cellular and network mechanisms. Neuron 62, 171–189. 10.1016/j.neuron.2009.04.008 19409263 PMC3132648

[B78] Hanley J. Henley J. (2005). PICK1 is a calcium-sensor for NMDA-induced AMPA receptor trafficking. EMBO J. 24, 3266–3278. 10.1038/sj.emboj.7600801 16138078 PMC1224691

[B79] Hebb D. O. (1949). Temperament in chimpanzees: method of analysis. J. Comp. Physiol. Psychol. 42, 192–206. 10.1037/h0056842 18151779

[B80] Heifets B. D. Castillo P. E. (2009). Endocannabinoid signaling and long-term synaptic plasticity. Annu. Rev. Physiol. 71, 283–306. 10.1146/annurev.physiol.010908.163149 19575681 PMC4454279

[B81] Henry F. E. McCartney A. J. Neely R. Perez A. S. Carruthers C. J. L. Stuenkel E. L.. (2012). Retrograde changes in presynaptic function driven by dendritic mTORC1. J. Neurosci. 32, 17128–17142. 10.1523/JNEUROSCI.2149-12.2012 23197706 PMC3518308

[B82] Hirsch J. C. Crepel F. (1990). Use-dependent changes in synaptic efficacy in rat prefrontal neurons *in vitro*. J. Physiol. 427, 31–49. 10.1113/jphysiol.1990.sp018159 2213602 PMC1189918

[B83] Hirsch J. C. Crepel F. (1991). Blockade of NMDA receptors unmasks a long-term depression insynaptic efficacy in rat prefrontal neurons *in vitro*. Exp. Brain Res. 85, 621–624. 10.1007/bf00231747 1680738

[B84] Hooper C. Markevich V. Plattner F. Killick R. Schofield E. Engel T.. (2007). Glycogen synthase kinase-3 inhibition is integral to long-term potentiation. Eur. J. Neurosci. 25, 81–86. 10.1111/j.1460-9568.2006.05245.x 17241269

[B85] Houston C. M. He Q. Smart T. G. (2009). CaMK II phosphorylation of the GABA_A_ receptor: receptor subtype- and synapse-specific modulation. J. Physiol. 587, 2115–2125. 10.1113/jphysiol.2009.171603 19332484 PMC2697286

[B86] Hsiung S. Tin A. Tamir H. Franke T. F. Liu K. (2008). Inhibition of 5-HT 1A receptor-dependent cell survival by cAMP/protein kinase A: role of protein phosphatase 2A and Bax. J. Neurosci. Res. 86, 2326–2338. 10.1002/jnr.21676 18459133

[B87] Huang F. S. Abbas A. K. Li R. Afanasenkau D. Wigström H. (2010). Bidirectional synaptic plasticity in response to single or paired pulse activation of NMDA receptors. Neurosci. Res. 67, 108–116. 10.1016/j.neures.2010.02.005 20170690

[B88] Huang Y. Y. Simpson E. Kellendonk C. Kandel E. R. (2004). Genetic evidence for the bidirectional modulation of synaptic plasticity in the prefrontal cortex by D1 receptors. Proc. Natl. Acad. Sci. U S A 101, 3236–3241. 10.1073/pnas.0308280101 14981263 PMC365773

[B89] Huemmeke M. Eysel U. T. Mittmann T. (2002). Metabotropic glutamate receptors mediate expression of LTP in slices of rat visual cortex. Eur. J. Neurosci. 15, 1641–1645. 10.1046/j.1460-9568.2002.02002.x 12059971

[B90] Isaacson J. S. Scanziani M. (2011). How inhibition shapes cortical activity. Neuron 72, 231–243. 10.1016/j.neuron.2011.09.027 22017986 PMC3236361

[B91] Jiang B. Huang S. de Pasquale R. Millman D. Song L. Lee H.-K.. (2010). The maturation of GABAergic transmission in visual cortex requires endocannabinoid-mediated LTD of inhibitory inputs during a critical period. Neuron 66, 248–259. 10.1016/j.neuron.2010.03.021 20435001 PMC2897012

[B92] Johnston D. Williams S. Jaffe D. Gray R. (1992). NMDA-receptor-independent long-term potentiation. Annu. Rev. Physiol. 54, 489–505. 10.1146/annurev.physiol.54.1.489 1314043

[B93] Johnstone V. P. A. Raymond C. R. (2013). Postsynaptic protein synthesis is required for presynaptic enhancement in persistent forms of long-term potentiation. Front. Synaptic Neurosci. 5:1. 10.3389/fnsyn.2013.00001 23450328 PMC3582942

[B94] Jones C. A. McCreary A. C. (2008). Serotonergic approaches in the development of novel antipsychotics. Neuropharmacology 55, 1056–1065. 10.1016/j.neuropharm.2008.05.025 18621404

[B95] Jope R. S. (2011). Glycogen synthase kinase-3 in the etiology and treatment of mood disorders. Front. Mol. Neurosci. 4:16. 10.3389/fnmol.2011.00016 21886606 PMC3152743

[B96] Kantrowitz J. Javitt D. C. (2012). Glutamatergic transmission in schizophrenia: from basic research to clinical practice. Curr. Opin. Psychiatry 25, 96–102. 10.1097/YCO.0b013e32835035b2 22297716 PMC5224527

[B97] Kasper S. Stein D. J. Loft H. Nil R. (2005). Escitalopram in the treatment of social anxiety disorder: randomised, placebo-controlled, flexible-dosage study. Br. J. Psychiatry 186, 222–226. 10.1192/bjp.186.3.222 15738503

[B98] Kehrer C. Maziashvili N. Dugladze T. Gloveli T. (2008). Altered excitatory-inhibitory balance in the NMDA-hypofunction model of schizophrenia. Front. Mol. Neurosci. 1:6. 10.3389/neuro.02.006.2008 18946539 PMC2525998

[B99] Kemp A. Manahan-Vaughan D. (2004). Hippocampal long-term depression and long-term potentiation encode different aspects of novelty acquisition. Proc. Natl. Acad. Sci. U S A 101, 8192–8197. 10.1073/pnas.0402650101 15150407 PMC419579

[B100] Kia H. K. Brisorgueil M. J. Hamon M. Calas A. Vergé D. (1996). Ultrastructural localization of 5-hydroxytryptamine1A receptors in the rat brain. J. Neurosci. Res. 46, 697–708. 10.1002/(sici)1097-4547(19961215)46:6<697::aid-jnr7>3.0.co;2-a 8978504

[B101] Kittler J. T. Moss S. J. (2003). Modulation of GABA_A_ receptor activity by phosphorylation and receptor trafficking: implications for the efficacy of synaptic inhibition. Curr. Opin. Neurobiol. 13, 341–347. 10.1016/s0959-4388(03)00064-3 12850219

[B102] Kleckner N. W. Dingledine R. (1988). Requirement for glycine in activation of NMDA-receptors expressed in Xenopus oocytes. Science 241, 835–837. 10.1126/science.2841759 2841759

[B103] Kruse M. S. Prémont J. Krebs M.-O. Jay T. M. (2009). Interaction of dopamine D1 with NMDA NR1 receptors in rat prefrontal cortex. Eur. Neuropsychopharmacol. 19, 296–304. 10.1016/j.euroneuro.2008.12.006 19186032

[B104] Lanzenberger R. R. Mitterhauser M. Spindelegger C. Wadsak W. Klein N. Mien L.-K.. (2007). Reduced serotonin-1A receptor binding in social anxiety disorder. Biol. Psychiatry 61, 1081–1089. 10.1016/j.biopsych.2006.05.022 16979141

[B106] Lemonde S. Turecki G. Bakish D. Du L. Hrdina P. D. Bown C. D.. (2003). Impaired repression at a 5-hydroxytryptamine 1A receptor gene polymorphism associated with major depression and suicide. J. Neurosci. 23, 8788–8879. 14507979 10.1523/JNEUROSCI.23-25-08788.2003 PMC6740417

[B105] Le Roux N. Amar M. Baux G. Fossier P. (2006). Homeostatic control of the excitation-inhibition balance in cortical layer 5 pyramidal neurons. Eur. J. Neurosci. 24, 3507–3518. 10.1111/j.1460-9568.2006.05203.x 17229099

[B107] Lewis D. A. Hashimoto T. Volk D. W. (2005). Cortical inhibitory neurons and schizophrenia. Nat. Rev. Neurosci. 6, 312–324. 10.1038/nrn1648 15803162

[B108] Li X. Rosborough K. M. Friedman A. B. Zhu W. Roth K. A. (2007). Regulation of mouse brain glycogen synthase kinase-3 by atypical antipsychotics. Int. J. Neuropsychopharmacol. 10, 7–19. 10.1017/s1461145706006547 16672106

[B110] Li Y.-C. Xi D. Roman J. Huang Y.-Q. Gao W.-J. (2009). Activation of glycogen synthase kinase-3 β is required for hyperdopamine and D2 receptor-mediated inhibition of synaptic NMDA receptor function in the rat prefrontal cortex. J. Neurosci. 29, 15551–15563. 10.1523/JNEUROSCI.3336-09.2009 20007479 PMC2832219

[B109] Li X. Zhu W. Roh M. S. Friedman A. B. Rosborough K. Jope R. S. (2004). *In vivo* regulation of glycogen synthase kinase-3β (GSK3β) by serotonergic activity in mouse brain. Neuropsychopharmacology 29, 1426–1431. 10.1038/sj.npp.1300439 15039769 PMC1986663

[B111] Lisman J. E. Zhabotinsky A. M. (2001). A model of synaptic memory: a CaMKII/PP1 switch that potentiates transmission by organizing an AMPA receptor anchoring assembly. Neuron 31, 191–201. 10.1016/S0896-6273(01)00364-6 11502252

[B112] Liu X. Y. Chu X. P. Mao L. M. Wang M. Lan H. X. Li M. H.. (2006). Modulation of D2R-NR2B interactions in response to cocaine. Neuron 52, 897–909. 10.1016/j.neuron.2006.10.011 17145509

[B113] Llano I. DiPolo R. Marty A. (1994). Calcium-induced calcium release in cerebellar Purkinje cells. Neuron 12, 663–673. 10.1016/0896-6273(94)90221-6 7512352

[B114] Lu B. (2003). BDNF and activity-dependent synaptic modulation. Learn. Mem. 10, 86–98. 10.1101/lm.54603 12663747 PMC5479144

[B115] Lucas-Meunier E. Monier C. Amar M. Baux G. Frégnac Y. Fossier P. (2009). Involvement of nicotinic and muscarinic receptors in the endogenous cholinergic modulation of the balance between excitation and inhibition in the young rat visual cortex. Cereb. Cortex 19, 2411–2427. 10.1093/cercor/bhn258 19176636

[B116] Łukasiewicz S. Błasiak E. Szafran-Pilch K. Dziedzicka-Wasylewska M. (2016). Dopamine D_2_ and serotonin 5-HT_1A_ receptor interaction in the context of the effects of antipsychotics–*in vitro* studies. J. Neurochem. 137, 549–560. 10.1111/jnc.13582 26876117

[B117] MacLean J. N. Watson B. O. Aaron G. B. Yuste R. (2005). Internal dynamics determine the cortical response to thalamic stimulation. Neuron 48, 811–823. 10.1016/j.neuron.2005.09.035 16337918

[B118] Malenka R. C. Nicoll R. A. (1999). Long-term potentiation–a decade of progress? Science 285, 1870–1874. 10.1126/science.285.5435.1870 10489359

[B119] Malinow R. Malenka R. C. (2002). AMPA receptor trafficking and synaptic plasticity. Annu. Rev. Neurosci. 25, 103–126. 10.1146/annurev.neuro.25.112701.14275 12052905

[B120] Malleret G. Alarcon J. M. Martel G. Takizawa S. Vronskaya S. Yin D.. (2010). Bidirectional regulation of hippocampal long-term synaptic plasticity and its influence on opposing forms of memory. J. Neurosci. 30, 3813–3825. 10.1523/JNEUROSCI.1330-09.2010 20220016 PMC6632240

[B121] Marco P. Sola R. G. Pulido P. Alijarde M. T. Sánchez A. Ramón y Cajal S.. (1996). Inhibitory neurons in the human epileptogenic temporal neocortex. An immunocytochemical study. Brain 119, 1327–1347. 10.1093/brain/119.4.1327 8813295

[B122] Marder C. P. Buonomano D. V. (2004). Timing and bbalanceof inhibition enhance the effect of long-term potentiation on cell firing. J. Neurosci. 24, 8873–8884. 10.1523/JNEUROSCI.2661-04.2004 15470154 PMC6729972

[B123] Marek G. J. (2007). Serotonin and dopamine interactions in rodents and primates: implications for psychosis and antipsychotic drug development. Int. Rev. Neurobiol. 78, 165–192. 10.1016/s0074-7742(06)78006-0 17349861

[B124] Markram H. Toledo-Rodriguez M. Wang Y. Gupta A. Silberberg G. Wu C. (2004). Interneurons of the neocortical inhibitory system. Nat. Rev. Neurosci. 5, 793–807. 10.1038/nrn1519 15378039

[B125] Marsicano G. Moosmann B. Hermann H. Lutz B. Behl C. (2002). Neuroprotective properties of cannabinoids against oxidative stress: role of the cannabinoid receptor CB1. J. Neurochem. 80, 448–456. 10.1046/j.0022-3042.2001.00716.x 11905991

[B126] Matsuda Y. Marzo A. Otani S. (2006). The presence of background dopamine signal converts long-term synaptic depression to potentiation in rat prefrontal cortex. J. Neurosci. 26, 4803–4810. 10.1523/JNEUROSCI.5312-05.2006 16672653 PMC6674173

[B127] McGuinness L. Taylor C. Taylor R. D. T. Yau C. Langenhan T. Hart M. L.. (2010). Presynaptic NMDARs in the hippocampus facilitate transmitter release at theta frequency. Neuron 68, 1109–1127. 10.1016/j.neuron.2010.11.023 21172613

[B128] Meisenzahl E. M. Schmitt G. Gründer G. Dresel S. Frodl T. la Fougère C.. (2008). Striatal D2/D3 receptor occupancy, clinical response and side effects with amisulpride: an iodine-123-iodobenzamide SPET study. Pharmacopsychiatry 41, 169–175. 10.1055/s-2008-1076727 18763218

[B130] Meltzer H. Y. Li Z. Kaneda Y. Ichikawa J. (2003). Serotonin receptors, their key role in drugs to treat schizophrenia. Prog. Neuropsychopharmacol. Biol. Psychiatry 27, 1159–1172. 10.1016/j.pnpbp.2003.09.010 14642974

[B131] Meltzer H. Y. Massey B. W. (2011). The role of serotonin receptors in the action of atypical antipsychotic drugs. Curr. Opin. Pharmacol. 11, 59–67. 10.1016/j.coph.2011.02.007 21420906

[B129] Meltzer C. C. Price J. C. Mathis C. A. Butters M. A. Ziolko S. K. Moses-Kolko E.. (2004). Serotonin 1A receptor binding and treatment response in late-life depression. Neuropsychopharmacology 29, 2258–2265. 10.1038/sj.npp.1300556 15483563

[B134] Meunier C. N. J. Amar M. Lanfumey L. Hamon M. Fossier P. (2013). 5-HT1A receptors direct the orientation of plasticity in layer 5 pyramidal neurons of the mouse prefrontal cortex. Neuropharmacology 71, 37–45. 10.1016/j.neuropharm.2013.03.003 23523560

[B133] Meunier C. N. Callebert J. Cancela J-M. Fossier P. (2015). Effect of dopaminergic D1 receptors on plasticity is dependent of serotoninergic 5-HT1A receptors in L5-pyramidal neurons of the prefrontal cortex. PLoS One 10:e0120286. 10.1371/journal.pone.0120286 25775449 PMC4361673

[B132] Meunier C. N. Cancela J. M. Fossier P. (2017). Lack of GSK3β activation and modulation of synaptic plasticity by dopamine in 5-HT1A-receptor KO mice. Neuropharmacology 113, 124–136. 10.1016/j.neuropharm.2016.09.025 27678414

[B135] Miller E. K. Cohen J. D. (2001). An integrative theory of prefrontal cortex function. Annu. Rev. Neurosci. 24, 167–202. 10.1146/annurev.neuro.24.1.167 11283309

[B136] Monier C. Chavane F. Baudot P. Graham L. J. Frégnac Y. (2003). Orientation and direction selectivity of synaptic inputs in visual cortical neurons: a diversity of combinations produces spike tuning. Neuron 37, 663–680. 10.1016/s0896-6273(03)00064-3 12597863

[B137] Monier C. Fournier J. Frégnac Y. (2008). *In vitro* and *in vivo* measures of evoked excitatory and inhibitory conductance dynamics in sensory cortices. J. Neurosci. Methods 169, 323–365. 10.1016/j.jneumeth.2007.11.008 18215425

[B138] Moreau A. W. Amar M. Le Roux N. Morel N. Fossier P. (2010). Serotoninergic fine-tuning of the excitation-inhibition balance in rat visual cortical networks. Cereb. Cortex 20, 456–467. 10.1093/cercor/bhp114 19520765

[B139] Mulkey R. M. Malenka R. C. (1992). Mechanisms underlying induction of homosynaptic long-term depression in area CA1 of the hippocampus. Neuron 9, 967–975. 10.1016/0896-6273(92)90248-c 1419003

[B140] Navailles S. Benazzouz A. Bioulac B. Gross C. De Deurwaerdère P. (2010). High-frequency stimulation of the subthalamic nucleus and L-3,4-dihydroxyphenylalanine inhibit *in vivo* serotonin release in the prefrontal cortex and hippocampus in a rat model of Parkinson’s disease. J. Neurosci. 30, 2356–2364. 10.1523/JNEUROSCI.5031-09.2010 20147561 PMC6634027

[B141] Negyessy L. Goldman-Rakic P. S. (2005). Subcellular localization of the dopamine D2 receptor and coexistence with the calcium-binding protein neuronal calcium sensor-1 in the primate prefrontal cortex. J. Comp. Neurol. 488, 464–475. 10.1002/cne.20601 15973684

[B142] Newman-Tancredi A. (2010). The importance of 5-HT1A receptor agonism in antipsychotic drug action, rationale and perspectives. Curr. Opin. Investig. Drugs 11, 802–812. 20571976

[B143] Newman-Tancredi A. Cussac D. Depoortere R. (2007). Neuropharmacological profile of bifeprunox: merits and limitations in comparison with other third-generation antipsychotics. Curr. Opin. Investig. Drugs 8, 539–554. 17659474

[B144] Newman-Tancredi A. Kleven M. S. (2011). Comparative pharmacology of antipsychotics possessing combined dopamine D2 and serotonin 5-HT1A receptor properties. Psychopharmacology (Berl) 216, 451–473. 10.1007/s00213-011-2247-y 21394633

[B145] Nicholls R. E. Alarcon J. M. Malleret G. Carroll R. C. Grody M. Vronskaya S.. (2008). Transgenic mice lacking NMDAR-dependent LTD exhibit deficits in behavioral flexibility. Neuron 58, 104–117. 10.1016/j.neuron.2008.01.039 18400167

[B146] Nowicky A. V. Bindman L. J. (1993). The nitric oxide synthase inhibitor, N-monomethyl-L-arginine blocks induction of a long-term potentiation-like phenomenon in rat medial frontal cortical neurons *in vitro*. J. Neurophysiol. 70, 1255–1259. 7693883 10.1152/jn.1993.70.3.1255

[B147] Nugent F. S. Penick E. C. Kauer J. A. (2007). Opioids block long-term potentiation of inhibitory synapses. Nature 446, 1086–1090. 10.1038/nature05726 17460674

[B148] Nyíri G. Stephenson F. A. Freund T. F. Somogyi P. (2003). Large variability in synaptic N-methyl-D-aspartate receptor density on interneurons and a comparison with pyramidal-cell spines in the rat hippocampus. Neuroscience 119, 347–363. 10.1016/s0306-4522(03)00157-x 12770551

[B149] Oleskevich S. Descarries L. (1990). Quantified distribution of the serotonin innervation in adult rat hippocampus. Neuroscience 34, 19–33. 10.1016/0306-4522(90)90301-j 2325849

[B150] Paspalas C. D. Goldman-Rakic P. S. (2005). Presynaptic D1 dopamine receptors in primate prefrontal cortex: target-specific expression in the glutamatergic synapse. J. Neurosci. 25, 1260–1267. 10.1523/JNEUROSCI.3436-04.2005 15689564 PMC6725972

[B151] Peineau S. Taghibiglou C. Bradley C. Wong T. P. Liu L. Lu J.. (2007). LTP inhibits LTD in the hippocampus via regulation of GSK3β. Neuron 53, 703–717. 10.1016/j.neuron.2007.01.029 17329210

[B152] Peters A. Kara D. (1985). The neuronal composition of area 17 of rat visual cortex. I. The pyramidal cells. J. Comp. Neurol. 234, 218–241. 10.1002/cne.902340208 3988983

[B153] Petersen C. C. Hahn T. T. Mehta M. Grinvald A. Sakmann B. (2003). Interaction of sensory responses with spontaneous depolarization in layer 2/3 barrel cortex. Proc. Natl. Acad. Sci. U S A 100, 13638–13643. 10.1073/pnas.2235811100 14595013 PMC263866

[B154] Pitler T. A. Alger B. E. (1992). Postsynaptic spike firing reduces synaptic GABAA responses in hippocampal pyramidal cells. J. Neurosci. 12, 4122–4132. 1403103 10.1523/JNEUROSCI.12-10-04122.1992 PMC6575956

[B155] Polter A. M. Li X. (2010). 5-HT1A receptor-regulated signal transduction pathways in brain. Cell. Signal. 22, 1406–1412. 10.1016/j.cellsig.2010.03.019 20363322 PMC2903656

[B156] Polter A. M. Yang S. Jope R. S. Li X. (2012). Functional significance of glycogen synthase kinase-3 regulation by serotonin. Cell. Signal. 24, 265–271. 10.1016/j.cellsig.2011.09.009 21946431 PMC3205250

[B157] Powell K. (2004). Opening a window to the autistic brain. PLoS Biol. 2:E267. 10.1371/journal.pbio.0020267 15314667 PMC509312

[B158] Puig M. V. Gulledge A. T. (2011). Serotonin and prefrontal cortex function: neurons, networks, and circuits. Mol. Neurobiol. 44, 449–464. 10.1007/s12035-011-8214-0 22076606 PMC3282112

[B159] Ramboz S. Oosting R. Amara D. A. Kung H. F. Blier P. Mendelsohn M.. (1998). Serotonin receptor 1A knockout: an animal model of anxiety-related disorder. Proc. Natl. Acad. Sci. U S A 95, 14476–14481. 10.1073/pnas.95.24.14476 9826725 PMC24398

[B160] Riad M. Garcia S. Watkins K. C. Jodoin N. Doucet E. Langlois X.. (2000). Somatodendritic localization of 5-HT1A and preterminal axonal localization of 5-HT1B serotonin receptors in adult rat brain. J. Comp. Neurol. 417, 181–194. 10.1002/(sici)1096-9861(20000207)417:2<181::aid-cne4>3.3.co;2-1 10660896

[B161] Rigas P. Castro-Alamancos M. A. (2007). Thalamocortical Up states: differential effects of intrinsic and extrinsic cortical inputs on persistent activity. J. Neurosci. 27, 4261–4272. 10.1523/JNEUROSCI.0003-07.2007 17442810 PMC6672324

[B162] Rippon G. Brock J. Brown C. Boucher J. (2007). Disordered connectivity in the autistic brain: challenges for the “new psychophysiology”. Int. J. Psychophysiol. 63, 164–172. 10.1016/j.ijpsycho.2006.03.012 16820239

[B163] Rubenstein J. L. (2010). Three hypotheses for developmental defects that may underlie some forms of autism spectrum disorder. Curr. Opin. Neurol. 23, 118–123. 10.1097/wco.0b013e328336eb13 20087182

[B164] Rubenstein J. L. R. Merzenich M. M. (2003). Model of autism: increased ratio of excitation/inhibition in key neural systems. Genes Brain Behav. 2, 255–267. 10.1034/j.1601-183x.2003.00037.x 14606691 PMC6748642

[B165] Saghatelyan A. K. Dityatev A. Schmidt S. Schuster T. Bartsch U. Schachner M. (2001). Reduced perisomatic inhibition, increased excitatory transmission and impaired long-term potentiation in mice deficient for the extracellular matrix glycoprotein tenascin-R. Mol. Cell. Neurosci. 17, 226–240. 10.1006/mcne.2000.0922 11161481

[B166] Saliba R. S. Michels G. Jacob T. C. Pangalos M. N. Moss S. J. (2007). Activity-dependent ubiquitination of GABA_A_ receptors regulates their accumulation at synaptic sites. J. Neurosci. 27, 13341–13351. 10.1523/JNEUROSCI.3277-07.2007 18045928 PMC6673389

[B167] Sanchez-Vives M. V. McCormick D. A. (2000). Cellular and network mechanisms of rhythmic recurrent activity in neocortex. Nat. Neurosci. 3, 1027–1034. 10.1038/79848 11017176

[B168] Santana N. Bortolozzi A. Serrats J. Mengod G. Artigas F. (2004). Expression of serotonin1A and serotonin2A receptors in pyramidal and GABAergic neurons of the rat prefrontal cortex. Cereb. Cortex 14, 1100–1109. 10.1093/cercor/bhh070 15115744

[B169] Santana N. Mengod G. Artigas F. (2009). Quantitative analysis of the expression of dopamine D1 and D2 receptors in pyramidal and GABAergic neurons of the rat prefrontal cortex. Cereb. Cortex 19, 849–860. 10.1093/cercor/bhn134 18689859

[B170] Sargent P. A. Kjaer K. H. Bench C. J. Rabiner E. A. Messa C. Meyer J.. (2000). Brain serotonin1A receptor binding measured by positron emission tomography with [11C]WAY-100635: effects of depression and antidepressant treatment. Arch. Gen. Psychiatry 57, 174–180. 10.1001/archpsyc.57.2.174 10665620

[B171] Shih J. C. (2004). Cloning, after cloning, knock-out mice, and physiological functions of MAO A and B. Neurotoxicology 25, 21–30. 10.1016/s0161-813x(03)00112-8 14697877

[B172] Shively C. A. Friedman D. P. Gage H. D. Bounds M. C. Brown-Proctor C. Blair J. B.. (2006). Behavioral depression and positron emission tomography-determined serotonin 1A receptor binding potential in cynomolgus monkeys. Arch. Gen. Psychiatry 63, 396–403. 10.1001/archpsyc.63.4.396 16585468

[B173] Shu Y. Hasenstaub A. Badoual M. Bal T. McCormick D. A. (2003a). Barrages of synaptic activity control the gain and sensitivity of cortical neurons. J. Neurosci. 23, 10388–10401. 14614098 10.1523/JNEUROSCI.23-32-10388.2003 PMC6741011

[B174] Shu Y. Hasenstaub A. McCormick D. A. (2003b). Turning on and off recurrent balanced cortical activity. Nature 423, 288–293. 10.1038/nature01616 12748642

[B175] Silberberg G. Markram H. (2007). Disynaptic inhibition between neocortical pyramidal cells mediated by Martinotti cells. Neuron 53, 735–746. 10.1016/j.neuron.2007.02.012 17329212

[B176] Singer H. S. Minzer K. (2003). Neurobiology of Tourette’s syndrome: concepts of neuroanatomic localization and neurochemical abnormalities. Brain Dev. 25, S70–S84. 10.1016/s0387-7604(03)90012-x 14980376

[B177] Sivakumaran S. Mohajerani M. H. Cherubini E. (2009). At immature mossy-fiber-CA3 synapses, correlated presynaptic and postsynaptic activity persistently enhances GABA release and network excitability via BDNF and cAMP-dependent PKA. J. Neurosci. 29, 2637–2647. 10.1523/JNEUROSCI.5019-08.2009 19244539 PMC6666235

[B178] Sjöström P. J. Turrigiano G. G. Nelson S. B. (2003). Neocortical LTD via coincident activation of presynaptic NMDA and cannabinoid receptors. Neuron 39, 641–654. 10.1016/s0896-6273(03)00476-8 12925278

[B179] Skinbjerg M. Namkung Y. Halldin C. Innis R. B. Sibley D. R. (2009). Pharmacological characterization of 2-methoxy-N-propylnorapomorphine’s interactions with D2 and D3 dopamine receptors. Synapse 63, 462–475. 10.1002/syn.20626 19217026 PMC2746040

[B180] Staff N. P. Spruston N. (2003). Intracellular corelate of EPSP-spike potentiation in CA1 pyramidal neurons is controlled by GABAergic modulation. Hippocampus 13, 801–805. 10.1002/hipo.10129 14620875

[B181] Stanton P. K. (1996). LTD, LTP, and the sliding threshold for long-term synaptic plasticity. Hippocampus 6, 35–42. 10.1002/(SICI)1098-1063(1996)6:1<35::AID-HIPO7>3.0.CO;2-6 8878740

[B182] Steinbusch H. W. (1981). Distribution of serotonin-immunoreactivity in the central nervous system of the rat—cell bodies and terminals. Neuroscience 6, 557–618. 10.1016/0306-4522(81)90146-9 7017455

[B183] Stephan K. E. Friston K. J. Frith C. D. (2009). Dysconnection in schizophrenia: from abnormal synaptic plasticity to failures of self-monitoring. Schizophr. Bull. 35, 509–527. 10.1093/schbul/sbn176 19155345 PMC2669579

[B184] Steriade M. Timofeev I. Grenier F. (2001). Natural waking and sleep states: a view from inside neocortical neurons. J. Neurophysiol. 85, 1969–1985. 11353014 10.1152/jn.2001.85.5.1969

[B185] Sutton L. P. Rushlow W. J. (2011). The effects of neuropsychiatric drugs on glycogen synthase kinase-3 signaling. Neuroscience 199, 116–124. 10.1016/j.neuroscience.2011.09.056 22001305

[B186] Timofeev I. Grenier F. Bazhenov M. Sejnowski T. J. Steriade M. (2000). Origin of slow cortical oscillations in deafferented cortical slabs. Cereb. Cortex 10, 1185–1199. 10.1093/cercor/10.12.1185 11073868

[B187] Topolnik L. Congar P. Lacaille J.-C. (2005). Differential regulation of metabotropic glutamate receptor- and AMPA receptor-mediated dendritic Ca^2+^ signals by presynaptic and postsynaptic activity in hippocampal interneurons. J. Neurosci. 25, 990–1001. 10.1523/JNEUROSCI.4388-04.2005 15673681 PMC6725617

[B188] Tseng K. Y. O’Donnell P. (2004). Dopamine-glutamate interactions controlling prefrontal cortical pyramidal cell excitability involve multiple signaling mechanisms. J. Neurosci. 24, 5131–5139. 10.1523/JNEUROSCI.1021-04.2004 15175382 PMC6729185

[B189] Turrigiano G. G. Nelson S. B. (2004). Homeostatic plasticity in the developing nervous system. Nat. Rev. Neurosci. 5, 97–107. 10.1038/nrn1327 14735113

[B190] Uylings H. B. M. Groenewegen H. J. Kolb B. (2003). Do rats have a prefrontal cortex? Behav. Brain Res. 146, 3–17. 10.1016/j.bbr.2003.09.028 14643455

[B192] Varela J. A. Hirsch S. J. Chapman D. Leverich L. S. Greene R. W. (2009). D1/D5 modulation of synaptic NMDA receptor currents. J. Neurosci. 29, 3109–3119. 10.1523/JNEUROSCI.4746-08.2009 19279248 PMC2684496

[B193] Wang X. Zhong P. Gu Z. Yan Z. (2003). Regulation of NMDA receptors by dopamine D4 signaling in prefrontal cortex. J. Neurosci. 23, 9852–9861. 14586014 10.1523/JNEUROSCI.23-30-09852.2003 PMC6740894

[B191] Van De Werd H. J. J. M. Rajkowska G. Evers P. Uylings H. B. M. (2010). Cytoarchitectonic and chemoarchitectonic characterization of the prefrontal cortical areas in the mouse. Brain Struct. Funct. 214, 339–353. 10.1007/s00429-010-0247-z 20221886 PMC2862954

[B194] Wehr M. Zador A. M. (2003). Balanced inhibition underlies tuning and sharpens spike timing in auditory cortex. Nature 426, 442–446. 10.1038/nature02116 14647382

[B195] White E. L. (1989). Cortical Circuits. Boston, MA: Birhäuser.

[B196] Wilsch V. W. Behnisch T. Jäger T. Reymann K. G. Balschun D. (1998). When are class I metabotropic glutamate receptors necessary for long-term potentiation? J. Neurosci. 18, 6071–6080. 9698302 10.1523/JNEUROSCI.18-16-06071.1998 PMC6793202

[B197] Woodgett J. R. (1990). Molecular cloning and expression of glycogen synthase kinase-3/factor A. EMBO J. 9, 2431–2438. 2164470 10.1002/j.1460-2075.1990.tb07419.x PMC552268

[B198] Woodgett J. R. (2001). Judging a protein by more than its name, GSK-3. Sci. STKE 2001:re12. 10.1126/stke.2001.100.re12 11579232

[B199] Xue M. Atallah B. V. Scanziani M. (2014). Equalizing excitation-inhibition ratios across visual cortical neurons. Nature 511, 596–600. 10.1038/nature13321 25043046 PMC4117808

[B201] Zhang Z. Jiao Y. Y. Sun Q. Q. (2011). Developmental maturation of excitation and inhibition balance in principal neurons across four layers of somatosensory cortex. Neuroscience 174, 10–25. 10.1016/j.neuroscience.2010.11.045 21115101 PMC3020261

[B202] Zheng P. Zhang X. X. Bunney B. S. Shi W. X. (1999). Opposite modulation of cortical N-methyl-D-aspartate receptor-mediated responses by low and high concentrations of dopamine. Neuroscience 91, 527–535. 10.1016/s0306-4522(98)00604-6 10366010

